# Pioneering an Innovative Eco‐Friendly N719 Dye‐Sensitized Solar Cell through Modelling and Impedance Spectroscopy Analysis for Energy Sustainability

**DOI:** 10.1002/gch2.202500276

**Published:** 2025-08-19

**Authors:** George G. Njema, Abderrahmane Elmelouky, Edson L. Meyer, Nassima Riouchi, Joshua K. Kibet

**Affiliations:** ^1^ Department of Chemistry Egerton University P.O Box 536 Egerton 20115 Kenya; ^2^ Laboratory Physics of Condensed Matter (LPMC) University of Chouaib Doukkali El‐Jadida 24000 Morocco; ^3^ University of Fort Hare Institute of Technology Alice 5700 South Africa; ^4^ Laboratory of Molecular Chemistry Materials and Environment Mohammed Premier University B.P. 300, Selouane Nador 62700 Morocco

**Keywords:** charge transport, electrical modulus, electrochemical performance, ion conduction, photovoltaic

## Abstract

Dye‐sensitized solar cells (DSSC) have received significant interest in the photovoltaic technology because of their eco‐friendly nature, affordability and flexibility. Here, this work presents a DSSC of the configuration; FTO/WO_3_/N719 Dye/GO/C with performance metrics – open‐circuit voltage (V_oc_) of 1.1055 V, short‐circuit current density (J_sc_) of 22.23 mA cm^−^^2^, a fill factor (FF) of 84.65%, and a remarkable power conversion efficiency (PCE) of 20.80%. The study utilizes a wide frequency range of 10^−3^ to 10^10^ Hz to examine charge transport dynamics and evaluate the electrochemical performance of the model cell. Impedance spectroscopy investigates both complex electrical impedance (Z*) and electric modulus (M*) to provide critical insights into ionic transport, charge recombination, ion migration and diffusion mechanisms within the cell. A model equivalent circuit is developed and theoretically validated by fitting experimental alternating current (AC) data to theoretical predictions, allowing the extraction of characteristic time constants for various processes. The results highlight that efficient ion conduction and rapid electron diffusion are essential for optimizing charge collection and minimizing recombination losses. Further, the study emphasizes the critical role of both series and shunt resistances across low‐ and high‐frequency domains, establishing a strong correlation between time constant behavior and overall device efficiency.

## Graphical abstract

Through rigorous simulations and analyses, a groundbreaking dye‐sanitized device architecture that integrates high photovoltaic performance with impressive power conversion efficiency of 20.8%., and utilizing affordable materials is proposed. Although the present cell architecture is investigated theoretically, the results inspires promise for design and engineering of practical dye sensitized solar cells for commercial applications.

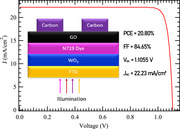

## 1 Introduction

Dye‐Sensitized Solar Cells (DSSCs), also known as Grätzel cells, are a groundbreaking innovations in the field of photovoltaic technology, offering a cost‐effective and environmentally friendly alternative to traditional silicon‐based solar cells.^[^ ^1^ ^]^ Invented in 1991 by Michael Grätzel and Brian O'Regan, DSSCs mimic the natural process of photosynthesis by using a photosensitive dye to absorb sunlight and generate electricity.^[^ ^2^ ^]^ This unique mechanism not only reduces the reliance on expensive and energy‐intensive manufacturing processes but also opens up new possibilities for scalable and sustainable solar energy solutions. Over the years, DSSCs have evolved significantly, with advancements in materials and design leading to improved efficiency, stability, and affordability. One of the most notable developments is the transition from liquid electrolytes to solid‐state materials, giving rise to solid‐state DSSCs, which address many of the limitations of earlier designs.^[^ ^3^ ^]^

The materials used in DSSCs and ssDSSCs (solid‐state dye‐sensitized solar cell) are central to their performance and affordability. Traditional DSSCs typically consist of a titanium dioxide (TiO_2_) photoanode, a ruthenium‐based dye sensitizer, a liquid electrolyte containing iodide/triiodide redox couples, and a platinum‐coated counter electrode.^[^ ^4^ ^]^ However, the shift to ssDSSCs has introduced solid hole‐transport materials such as spiro‐OMeTAD, conductive polymers, and inorganic compounds, which enhance stability and durability.^[^ ^5^ ^]^ Moreover, the use of carbon‐based materials, metal‐organic frameworks (MOFs), and quantum dots has further improved efficiency while reducing costs.^[^ ^6^ ^]^ These advancements in materials science have made DSSCs more accessible and economically viable, particularly for applications in off‐grid and rural electrification.

Accordingly, this study presents a newly designed ssDSSC with an impressive photoconversion efficiency. The novelty of this design can be ascribed to the unique combination of tungsten trioxide (WO_3_) as the electron transport layer (ETL) and graphene oxide (GO) as the hole transport layer (HTL). The present cell design, represents an innovative approach to ssDSSCs, addressing several key challenges in the field, such as cost, stability, and efficiency. Each layer in this configuration is critical in the functionality of the device, and the choice of materials reflects a balance between performance and affordability. The configuration comprises fluorine‐doped tin oxide (FTO), which serves as the transparent conducting oxide (TCO) layer. FTO acts as the front electrode, allowing light to pass through while providing excellent electrical conductivity.^[^ ^7^ ^]^ With a wide band gap of ≈3.5–4.0 eV, FTO is transparent to visible light,^[^ ^8^ ^]^ making it ideal for solar cell applications. Its high electron mobility ensures efficient charge collection, while its relatively low cost and widespread availability make it a practical choice for large‐scale applications.^[^ ^9^ ^]^ The inclusion of FTO addresses the need for a low‐resistance, transparent electrode, which is essential for maximizing light absorption and minimizing energy losses in ssDSSCs. Tungsten trioxide (WO_3_) is employed as the electron transport layer (ETL). WO_3_ plays a crucial role in extracting electrons from the photoexcited dye and transporting them to the FTO electrode.^[^ ^10^ ^]^ With a band gap of ≈2.6–2.8 eV, WO_3_ can absorb visible light,^[^ ^11^ ^]^ contributing to the overall light‐harvesting capability of the device. Its moderate electron mobility (≈10 cm^2^ Vs^−1^) and good electrical conductivity make it an effective ETL material.^[^ ^12^ ^]^ Additionally, WO_3_ is relatively inexpensive and abundant, offering a cost‐effective alternative to traditional materials like titanium dioxide (TiO_2_). Using carbon as the back electrode (work function of 5.0 eV)^[^ ^13^ ^]^ has effectively enhanced charge extraction, suppressed recombination losses, and markedly lowered the overall fabrication expenses.

To address the challenge of hole transport layer (HTL), graphene oxide (GO) is incorporated. Here, GO facilitates the extraction of holes from the dye and transports them to the counter electrode. Its band gap, which is tunable between 1.7 and 2.4 eV depending on the degree of oxidation,^[^ ^14^ ^]^ allows for flexibility in optimizing its electronic properties. GO exhibits high hole mobility and moderate to high conductivity, making it an excellent HTL material.^[^ ^15^ ^]^ Moreover, GO is relatively affordable compared to other HTL materials like spiro‐OMeTAD, which are often expensive and complex to synthesize.^[^ ^16^ ^]^ The inclusion of GO in this configuration addresses the need for a low‐cost, efficient HTL that can improve charge transport and reduce recombination, thereby enhancing the stability and efficiency of ssDSSCs. Ultimately, carbon (C) is used as the counter electrode, completing the electrical circuit by collecting holes from the HTL and facilitating the reduction of the electrolyte (if present). Carbon is a highly conductive material, enabling efficient charge collection. Its exceptional carrier mobility (≈10 000 cm^2^ Vs^−1^ for graphene oxide) and low cost make it an ideal replacement for expensive metal‐based counter electrodes.^[^ ^17^ ^]^ The use of carbon addresses the need for a scalable and cost‐effective counter electrode material, further reducing the overall cost of the device while maintaining high performance.

N719 dye, a ruthenium‐based sensitizer, acts as the photoactive layer which absorbs photon energy and produces electron‐hole pairs.^[^ ^18^ ^]^ With a band gap of ≈1.7–1.8 eV, the N719 dye efficiently captures visible light, making it a highly effective photosensitizer.^[^ ^19^ ^]^ However, its reliance on ruthenium, a rare and expensive metal, increases the overall cost of the device. Despite this drawback, the N719 dye is indispensable for its relatively high efficiency and broad absorption spectrum, which are critical for the operation of ssDSSCs.^[^ ^20^ ^]^ Ruthenium‐based dyes such as N719 have long been established as benchmark sensitizers in DSSCs, including their solid‐state counterparts. Their widespread use is largely attributed to their excellent photochemical stability, reliable performance, and well‐understood charge transfer dynamics. Despite the fact that N719 effectively captures light in the visible region, it does not efficiently harvest photons in the near‐infrared (NIR) region, thereby limiting the overall light‐harvesting capability of the device.^[^ ^21^ ^]^ Moreover, the relatively high cost and limited availability of ruthenium raise concerns about the economic viability and environmental sustainability of large‐scale applications. The synthetic complexity of preparing ruthenium dyes further adds to the cost and manufacturing challenges. Nevertheless, N719 maintains strong compatibility with mesoporous TiO_2_ photoanodes and commonly used HTLs like Spiro‐OMeTAD.^[^ ^22^ ^]^ It facilitates efficient electron injection into the conduction band of TiO_2_ and demonstrates good resistance to charge recombination, ascribed to its anchoring groups and molecular structure that offer steric hindrance. These features contribute to respectable fill factors and relatively stable operation.

Although N719 dye displays good stability under low‐temperature light exposure, it has been known to degrade considerably under high temperature soaking leading to decreased photovoltaic parameters – open‐circuit voltage (Voc), short‐circuit current (Jsc), and overall power conversion efficiency (PCE).^[^ ^23^ ^]^ Various strategies can be used to enhance the stability of the N719 dye including tandem dye sensitized solar cell architecture and co‐sensitization such as Cu‐doped CdS which not only enhances stability but also improves light capturing ability.^[^ ^24^, ^25^ ^]^ Further, machine learning and artificial intelligence offers promise in understanding degradation phenomena in dye sensitized‐based solar cells for better engineered cell architectures and material selection.^[^ ^26^ ^]^ In terms of photovoltaic performance, N719 typically achieves practical PCEs in the range of 7.6 to 9.8% under solid‐state configurations.^[^ ^27^, ^28^ ^]^ While this was once considered impressive, it faces competition with recent developments in the field, particularly those involving metal‐free organic dyes and perovskite materials. Organic dyes such as D35 or C106 have shown comparable or slightly better efficiencies more than 9% and offer the advantage of being metal‐free, less toxic and potentially more cost‐effective.^[^ ^29^ ^]^ On the other hand, tandem dye‐sensitized solar cells (T‐DSSCs) based on organic dyes have reported practical photoconversion efficiencies exceeding 12%.^[^ ^30^ ^]^ A study by Shao and Wu (2022) reported a PCE of 10.26% using N719 dye tandem configuration.^[^ ^31^ ^]^ Also, porphyrin‐based dye YD2‐o‐C8 has demonstrated remarkable performance, achieving up to 12.5% efficiency, due to its broad spectral absorption and excellent charge transport, but using a rather expensive HTL (Spiro‐OMeTAD).^[^ ^32^, ^33^ ^]^

The selection of an appropriate sensitizing dye is a key factor influencing the overall efficiency and stability of DSSCs. Among the various ruthenium‐based dyes developed, N719 has emerged as one of the most reliable and high‐performing options. Compared to other dyes in the same class, such as N_3_ and Black Dye, N719 consistently delivers better device performance due to its favorable light absorption characteristics, chemical stability, and strong binding affinity to TiO_2_ surfaces. In contrast, natural dyes derived from sources like anthocyanins (from berries), chlorophyll (from spinach), and betalains (from beetroot) offer environmentally friendly alternatives with low toxicity and simple extraction methods.^[^ ^34^ ^]^ However, these dyes typically suffer from limited absorption ranges, poor long‐term stability, and lower power conversion efficiencies generally not exceeding 2%.^[^ ^35^ ^]^ Moreover, their performance often varies significantly depending on extraction conditions and device fabrication methods. When benchmarked against newer classes of sensitizers, N719 still offers a compelling balance between efficiency and reliability. Organic dyes, while more sustainable and synthetically tunable, often face limitations in photochemical stability and reproducibility across different fabrication processes.^[^ ^34^ ^]^

Natural dyes have become increasingly popular in DSSCs because they are environmentally friendly, affordable, and easy to extract.^[^ ^20^, ^36^ ^]^ These dyes, derived from plants, fruits, and flowers, serve as sensitizers in DSSCs. Anthocyanins, for example, are commonly extracted from blueberries, blackberries, and red cabbage and are valued for their ability to absorb visible light.^[^ ^37^ ^]^ Chlorophyll, obtained from spinach or other green leaves, is another widely used dye, as it replicates the natural process of photosynthesis.^[^ ^38^ ^]^ Other natural dyes, such as betalains from beetroot and carotenoids from carrots or marigold flowers, are also employed for their light‐absorbing properties.^[^ ^39^ ^]^ Additionally, curcumin from turmeric and tannins from tea leaves have demonstrated potential as effective sensitizers.^[^ ^40^ ^]^ While natural dyes provide a sustainable alternative to synthetic ones, their efficiency in DSSCs tends to be lower. Researchers are actively working to enhance their performance by refining extraction techniques, improving light absorption, and optimizing DSSC designs to boost energy conversion rates.

DSSCs contribute significantly to the United Nations Sustainable Development Goals (SDGs), particularly Goal 7 (Affordable and Clean Energy) and Goal 13 (Climate Action).^[^ ^41^, ^42^ ^]^ By providing a low‐cost and renewable energy source, DSSCs may offer promise to reduce global reliance on fossil fuels and mitigate greenhouse gas emissions. Their ability to operate efficiently under low‐light conditions and diffuse sunlight makes them ideal for regions with limited access to reliable energy. Furthermore, the use of eco‐friendly and abundant materials in ssDSSCs aligns with Goal 12 (Responsible Consumption and Production), promoting sustainable industrial practices and a circular economy. This alignment with global sustainability goals underscores the importance of DSSCs in addressing both energy poverty and environmental degradation.

The adoption of DSSCs also has profound implications for green energy and carbon footprint reduction.^[^ ^43^ ^]^ Unlike conventional solar cells, which require high‐temperature processing and rare materials, DSSCs can be manufactured using low‐cost and abundant resources, significantly reducing their energy payback time and overall carbon emissions.^[^ ^43^ ^]^ The lightweight and flexible nature of ssDSSCs further expands their applications, enabling integration into building materials, vehicles, and portable devices.^[^ ^5^, ^44^ ^]^ This versatility not only enhances their utility but also supports the global transition to renewable energy. By harnessing solar energy more efficiently and sustainably, DSSCs play a crucial role in combating climate change and achieving net‐zero emissions.^[^ ^45^ ^]^ One of the most compelling aspects of DSSCs is their affordability, which makes them accessible to a broader range of consumers, including those in developing countries. The use of affordable materials including organic dyes, carbon‐based counter electrodes, and conductive polymers has significantly reduced production costs.^[^ ^46^ ^]^ Additionally, the modular design of DSSCs allows for easy installation and maintenance, further lowering the barriers to adoption. This affordability is particularly important for off‐grid and rural communities, where access to reliable and clean energy remains a challenge. By democratizing access to solar energy, DSSCs empower communities to achieve energy independence and improve their quality of life, while also contributing to global efforts to reduce energy inequality.^[^ ^47^ ^]^

Despite their numerous advantages, DSSCs face challenges that must be addressed to realize their full potential. Issues such as durability, efficiency, scalability and stability under varying environmental conditions remain areas of active research. For instance, perovskite‐based ssDSSCs, while highly efficient, are susceptible to degradation from moisture and heat. Researchers are exploring encapsulation techniques and alternative materials to enhance durability and performance. Collaborative efforts between academia, industry, and policymakers are essential to accelerate the commercialization and deployment of DSSCs. With continued innovation and investment, DSSCs have the potential to revolutionize the solar energy landscape and contribute significantly to a sustainable energy future.^[^ ^48^ ^]^

This study introduces a transformative advancement in solar energy technology through a novel DSSC design that harmonizes efficiency with environmental sustainability. Advanced computational modelling via SCAPS‐1D simulations elucidates the mechanisms behind suppressed charge recombination and improved carrier mobility, providing a universal blueprint for future sustainable photovoltaic development. This work bridges critical gaps between industrial scalability, cost‐effectiveness, and ecological responsibility, positioning DSSCs as a cornerstone technology for global renewable energy transitions. The non‐destructive and easily applicable nature of impedance spectroscopy (IS) for evaluating systems has led to its widespread adoption in numerous fields for characterizing materials. Both industrial and scientific communities are increasingly acknowledging the value of IS, especially in applications related to electrocatalysis and energy,^[^ ^49^, ^50^ ^]^ in evaluating the quality of coatings,^[^ ^51^ ^]^ in monitoring corrosion phenomena,^[^ ^52^, ^53^ ^]^ and in the evaluation and adjustment of sensors.^[^ ^54^, ^55^ ^]^

This study addresses dye degradation by implementing a solid‐state design that replaces the conventional liquid electrolyte with a stable solid HTL (GO) and employs carbon as the counter electrode. This configuration significantly reduces the exposure of the dye to volatile or corrosive media, enhancing both thermal and photostability. Furthermore, the use of carbon as a counter electrode, with its high conductivity, chemical stability, and affordability, eliminates the need for expensive and degradation‐prone metallic contacts. WO_3_ offers a favorable conduction band alignment with the dye and FTO, which promotes the effective extraction of electrons from the photoexcited dye and their transport toward the external circuit. Its moderate electron mobility and ability to absorb visible light contribute to enhanced electron collection and light‐harvesting capabilities.^[^ ^56^ ^]^ This makes WO_3_ a valuable alternative to more conventional ETLs such as TiO_2_, particularly in terms of its conductivity and compatibility with visible‐light‐active dyes. To this end, the choice of WO_3_, GO, and carbon, along with the transition to a solid‐state configuration, offers a promising pathway for the development of robust, efficient, and cost‐effective dye‐sensitized solar technologies for promising high performance, stability and scalability. Although the present cell architecture is investigated theoretically, the results offer insights into the device performance and inspires future fabrication of a high‐performance physical cell.

## 2 Device Architecture and Simulation

Solar Cell Capacitance Simulator (SCAPS‐1D) is a widely used software for simulating and analyzing the performance of solar cells,^[^ ^57^, ^58^, ^59^ ^]^ particularly thin‐film technologies such as CIGS (Copper Indium Gallium Selenide), CdTe (Cadmium Telluride), ssDSSC and perovskite‐based devices. The software provides a comprehensive platform for evaluating the electrical and optical properties of solar cells under different conditions, making it a valuable tool for researchers and engineers seeking to optimize device performance.^[^ ^57^ ^]^

At the core of SCAPS‐1D is its ability to model carrier transport by solving key semiconductor equations, including Poisson's equation as shown in Equation 1.^[^ ^60^ ^]^

(1) $$\frac{d}{d_{x}}\left(-\varepsilon \left(x\right)\frac{d\psi }{dx}\right)=q\left[p\left(x\right)-n\left(x\right)+{N^{+}}_{d}\left(x\right)-{N^{-}}_{a}\left(x\right)\right]$$

Here, ɛ denotes the permittivity denotes the electron charge, ψ denotes the electrostatic potential, n is the total electron density, prepresents thetotalholedensity, *N* ^+^_*d*_represents the ionized donor‐like doping concentration, and *N* ^−^_*a*_ denotes the ionized acceptor‐like doping concentration.

Continuity equations for electrons and holes, and drift‐diffusion equations as shown in Equation 2.^[^ ^60^ ^]^

(2) $$\frac{1}{q}\left(\frac{dJ_{P}}{dx}\right)=G_{OP}\left(x\right)-R\left(x\right)$$

where *J_p_* is denoted by the electron and hole current densities, respectively. The parameter *R*(*x*)is used for the net recombination rate.

This capability allows users to analyze charge carrier behavior within the device and assess its impact on efficiency. Additionally, SCAPS‐1D incorporates various recombination mechanisms that influence solar cell performance.^[^ ^61^ ^]^ By defining these mechanisms, researchers can better understand and mitigate energy losses within the device. Another important feature of SCAPS‐1D is its ability to model defect states both within the bulk material and at interfaces. Parameters such as defect energy levels, capture cross‐sections, and defect densities can be customized, enabling the study of how these imperfections affect exciton dynamics and overall device performance. The software also allows users to define illumination conditions by specifying the spectrum of incident light, such as the AM 1.5G solar spectrum under one sun at 1000 W M^−2^,^[^ ^62^ ^]^ and calculating the optical generation of electron‐hole pairs based on the absorber layer's absorption profile.

In addition to optical and electrical modelling, SCAPS‐1D supports bias condition simulations, including voltage sweep and capacitance‐voltage (C‐V) analysis.^[^ ^63^ ^]^ The voltage sweep function is essential for generating current‐voltage (J‐V) characteristics, which are crucial for determining key performance metrics^[^ ^64^ ^]^ such as power conversion efficiency (PCE), fill factor (FF), short‐circuit current density (J_sc_), and open‐circuit voltage (V_oc_). Meanwhile, C‐V analysis helps investigate doping profiles and defect states, providing deeper insights into the electrical behavior of the solar cell.^[^ ^65^ ^]^Overall, SCAPS‐1D is a powerful and user‐friendly simulation tool that offers detailed insights into the electrical and optical behavior of solar cells. Despite some limitations, its versatility and ease of use make it an essential resource for optimizing solar cell performance and guiding experimental research.

**Figure 1:**
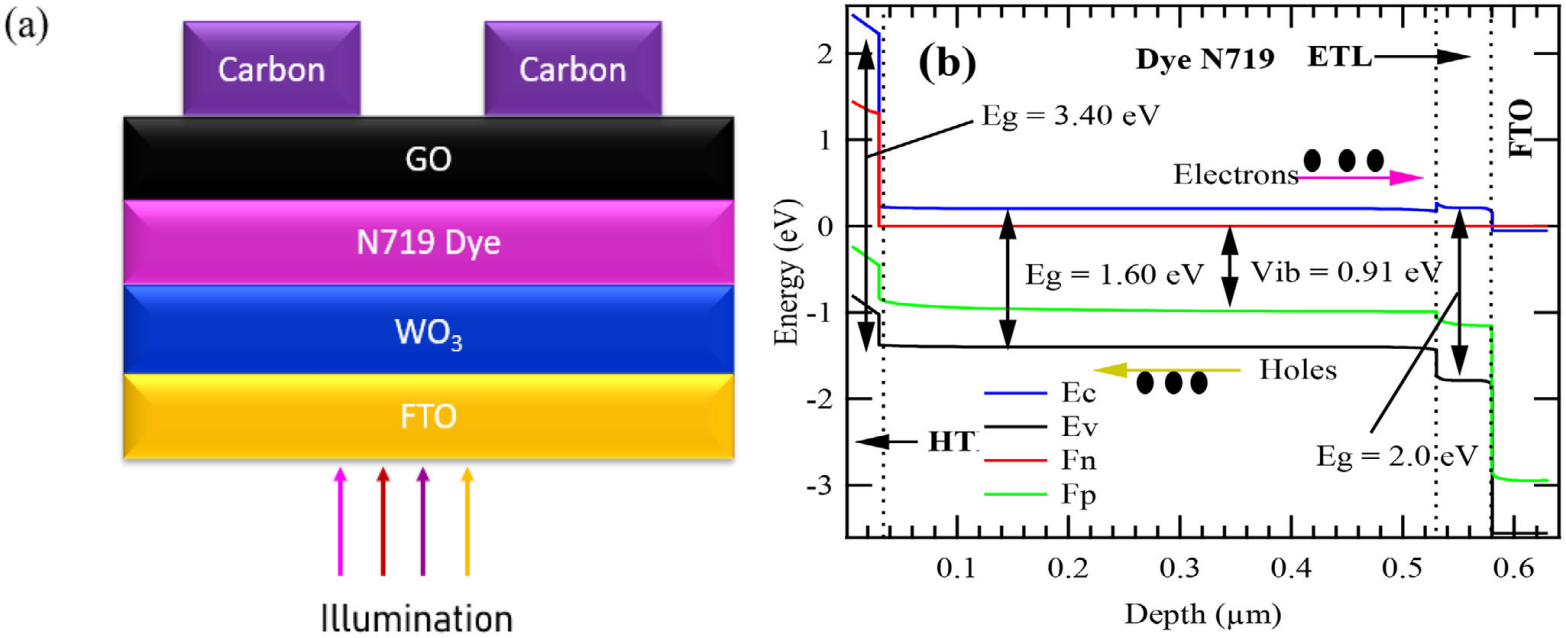
The proposed solar cell's structure (a) and (b), the corresponding energy levels band diagrams.

**Figure** **1**a illustrates the configuration and linking of individual photovoltaic cells within a module, which influences the voltage, current, and power output of the solar panel. Figure 1b provides a comprehensive overview of the energy levels within the proposed photovoltaic device. This diagram is essential for understanding the electronic processes that facilitate the charge carrier transport, which are critical for the device's operation. At the core of this diagram are the conduction band (CB) and valence band (VB). The conduction band represents the range of energy levels where electrons can move freely, enabling electrical conduction.^[^ ^66^ ^]^ In contrast, the valence band consists of lower energy levels where electrons are bound to atoms and do not contribute to conduction.^[^ ^67^ ^]^ The energy difference between these bands is a fundamental aspect of the material's electronic properties. The illustration further points out the Highest Occupied Molecular Orbital (HOMO) and the Lowest Unoccupied Molecular Orbital (LUMO). At absolute zero, the HOMO represents the uppermost energy level filled with electrons, and the LUMO is the lowest available energy level for electron occupancy.^[^ ^68^ ^]^ These molecular orbitals are crucial in determining how electrons transition between different states within the system.^[^ ^69^ ^]^

Figure 1b is the band energy level diagram which represents the electronic structure of the materials, highlighting the valence band, conduction band, and the bandgap.^[^ ^57^, ^58^ ^]^ When sunlight with energy exceeding the bandgap strikes the semiconductor, photons are absorbed, promoting electrons from the valence band to the conduction band to generate excitons.^[^ ^70^ ^]^ The diagram also shows the built‐in electric field at the p‐n junction, which separates these charge carriers, directing electrons to the n‐type region and holes to the p‐type region, thereby producing a photocurrent.

The energy levels within the device are crucial for understanding its operation. The conduction band edge (Ec) represents the lowest unoccupied energy level where electrons can move freely, while the valence band edge (Ev) signifies the highest occupied energy level where holes can move freely. In the diagram, the band gaps are specified for different materials: 3.40 eV for the ETL (WO_3_), 1.60 eV for the dye, and 2.0 eV for the HTL. Additionally, the electron quasi‐Fermi level (Fn) and the hole quasi‐Fermi level (Fp) represent the electrochemical potentials of electrons and holes, respectively, with their positions indicating the population of these charge carriers. The built‐in potential (Vib), which is the difference between the Fermi levels of the FTO and the HTL at equilibrium, is measured at 0.91 eV, reflecting the potential difference across the device. Generally, Figure 1b serves as a comprehensive visual representation of the energy band structure and charge transport mechanisms in a DSSC. It underscores the importance of careful material selection, precise interface engineering, and optimal energy level alignment to achieve efficient solar energy conversion. **Tables** **1** and **2** present the optimized and the initial input parameters, respectively.

**Table 1:** Input parameters of the proposed optimized solar cell.

Parameter	GO^[^ ^71^, ^72^ ^]^	N719 dye^[^ ^72^ ^]^	WO_3_^[^ ^73^ ^]^	FTO^[^ ^74^, ^75^ ^]^
Thickness, (nm)	30.00	500.00	50.00	50.00
Bandgap, E_g_, (eV)	3.25	1.6	2.0	3.5
Electron affinity, χ, (eV)	1.9	3.9	3.8	4
Dielectric permittivity, relative, (ɛ_r_)	3	30	4.8	9
CB effective density of states, N_c_, (cm^−3^)	2.2 × 10^21^	2.4 × 10^20^	2.2 × 10^21^	2.2 × 10^18^
VB effective density of state, N_v,_ (cm^−3^)	1.8 × 10^19^	2.5 × 10^20^	2.2 × 10^21^	1.8 × 10^19^
Electron mobility, µ_n_, (cm^2^ V^−1^S^−1^)	1.0 × 10^2^	5.0 × 10^0^	3.0 × 10^1^	2.0 × 10^1^
Hole mobility, µ_p_, (cm^2^ V^−1^S^−1^)	3.0 × 10^2^	5.0 × 10^0^	3.0 × 10^1^	1.0 × 10^1^
Density of n‐type doping, N*_D_*, (cm^−3^)	1.0 × 10^16^	1.0 × 10^17^	6.35 × 10^17^	2.0 × 10^19^
Density of p‐type doping, N_A_, (cm^−3^)	1.0 × 10^16^	1.0 × 10^7^	0.0 × 10^0^	0.0 × 10^0^
Defect density, N_t_(cm^−3^)	1.0 × 10^14^	1.0 × 10^14^	1.0 × 10^15^	1.0 × 10^15^

**Table 2:** Initial input parameters of the proposed cell structure.

Parameter	GO^[^ ^72^ ^]^	N719 dye^[^ ^72^, ^74^ ^]^	WO_3_	FTO^[^ ^74^, ^75^ ^]^
Thickness, (nm)	200	200	10	280
Bandgap, E_g_, (eV)	3.25	1.6	2.0	3.5
Electron affinity, χ, (eV)	1.9	3.9	3.8	4
Dielectric permittivity, relative, (ɛ_r_)	3	30	4.8	9
CB effective density of states, N_c_, (cm^−3^)	2.2 × 10^21^	2.4 × 10^20^	2.2 × 10^21^	2.2 × 10^18^
VB effective density of state, N_v,_ (cm^−3^)	1.8 × 10^19^	2.5 × 10^20^	2.2 × 10^21^	1.8 × 10^19^
Electron mobility, µ_n_, (cm^2^ V^−1^S^−1^)	1.0 × 10^2^	5.0 × 10^0^	3.0 × 10^1^	2.0 × 10^1^
Hole mobility, µ_p_, (cm^2^ V^−1^S^−1^)	3.0 × 10^2^	5.0 × 10^0^	3.0 × 10^1^	1.0 × 10^1^
Density of n‐type doping, N*_D_*, (cm^−3^)	1.0 × 10^16^	1.0 × 10^17^	6.35 × 10^17^	2.0 × 10^19^
Density of p‐type doping, N_A_, (cm^−3^)	1.0 × 10^16^	1.0 × 10^7^	0.0 × 10^0^	0.0 × 10^0^
Defect density, N_t_(cm^−3^)	1.0 × 10^14^	1.0 × 10^14^	1.0 × 10^15^	1.0 × 10^15^

## 3 Results and Discussion

### 3.1 J‐V and Q‐E Characteristics for the Initial and the Optimized Device

**Figure 2:**
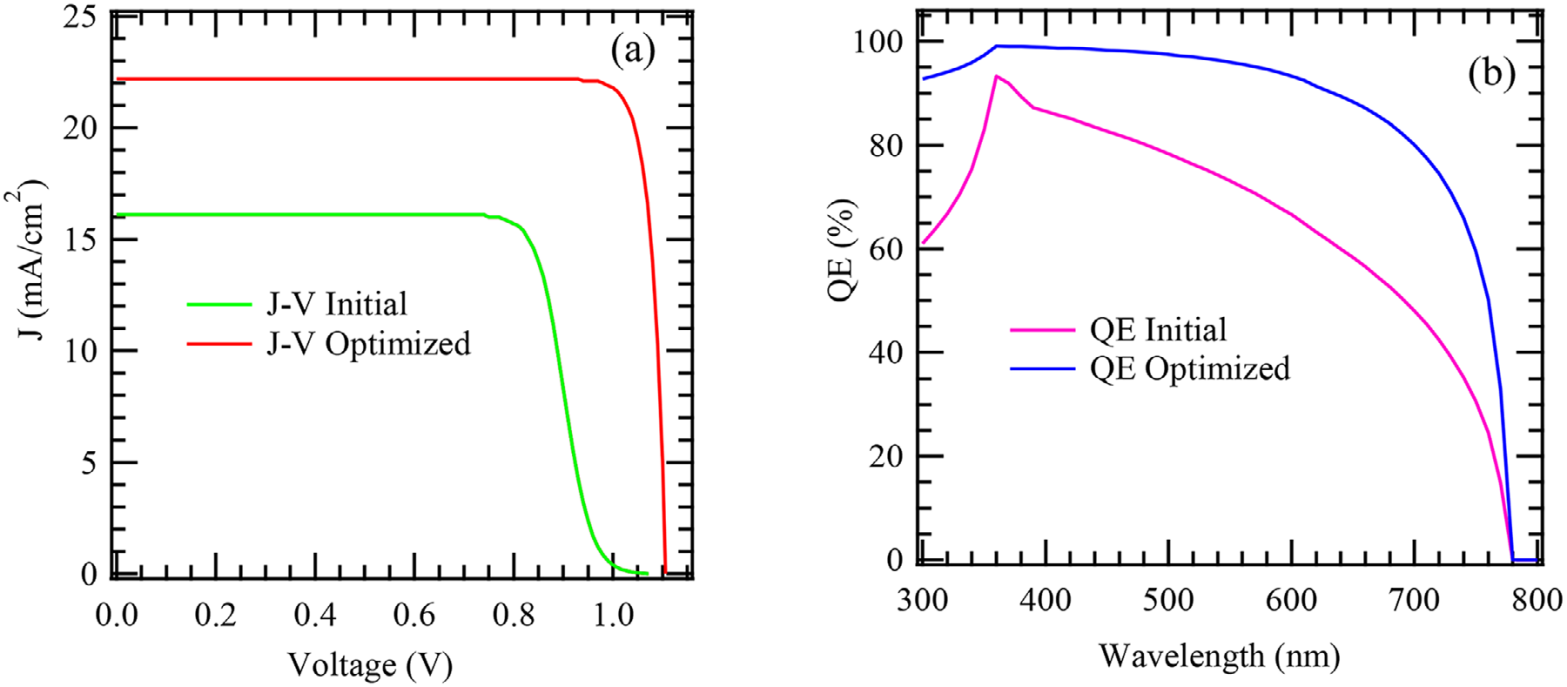
a) J‐V and b) QE plots for the initial and the optimized cell structure.

**Figure** **2**a illustrates the J–V characteristics of the model solar cell structure before and after optimization. A noticeable enhancement is observed in key photovoltaic parameters – J_sc_, V_oc_, FF, and PCE. The improved curve shape after optimization is marked by a higher plateau and a sharper transition near V_oc_ suggesting better charge transport, reduced recombination, and more efficient energy level alignment. These improvements likely result from refined device architecture, such as optimized layer thicknesses, improved band alignment.

On the other hand, Figure 2b shows the variation in Quantum Efficiency (QE) of the model device across different wavelengths, comparing performance before and after optimization. The optimized device exhibits significantly enhancement in QE throughout the active spectral range, particularly between 400 and 800 nm, highlighting increased light absorption and charge carrier collection. The sharp decline beyond 800 nm corresponds to the material's absorption limit. Overall, the results demonstrate that the optimization improves the photo response and efficiency of the device. The summarized photovoltaic outcomes for the initial and optimized cell structures are presented in **Table** **3**.

**Table 3:** Performance metrics of J‐V before and after optimization of the model cell structure.

	V_oc_ [V]	J_sc_ [mA cm^−2^]	FF [%]	PCE [%]
Initial	1.0717	16.12	73.07	12.62
After Optimization	1.1055	22.23	84.65	20.80

### 3.2 The Absorption Model of the Cell Structure

Optimizing light harvesting and charge generation in ssDSSCs heavily relies on the absorption model. In ssDSSCs, light absorption is influenced by the thickness of the dye, and the optical properties of the surrounding materials.^[^ ^36^ ^]^ A well‐optimized absorption model accounts for these factors to ensure maximum photon capture and minimal energy losses. The dye's absorption coefficient and its ability to cover a broad spectral range directly impact photocurrent generation.^[^ ^1^ ^]^ Unlike liquid‐based DSSCs, where electrolyte penetration affects absorption, ssDSSCs utilize solid‐state HTLs, requiring careful optimization of their optical and electronic properties to enhance light absorption and charge transport.

Furthermore, the absorption model considers internal reflections and interference effects within the device structure. Light management strategies, such as plasmonic enhancement, textured electrodes, and photonic crystals, can be employed to improve absorption efficiency.^[^ ^76^ ^]^ The choice of HTL, typically an organic or inorganic semiconductor, influences optical transparency and refractive index matching, which affect overall absorption characteristics.^[^ ^77^ ^]^ Advanced modelling techniques, including transfer matrix methods and finite‐difference time‐domain (FDTD) simulations,^[^ ^78^, ^79^ ^]^ enable researchers to optimize the absorption profile by predicting light propagation in the multilayer structure of ssDSSCs. By refining the absorption model, the PCE of ssDSSCs can be significantly improved, making them more competitive with conventional silicon‐based photovoltaics.

**Figure 3:**
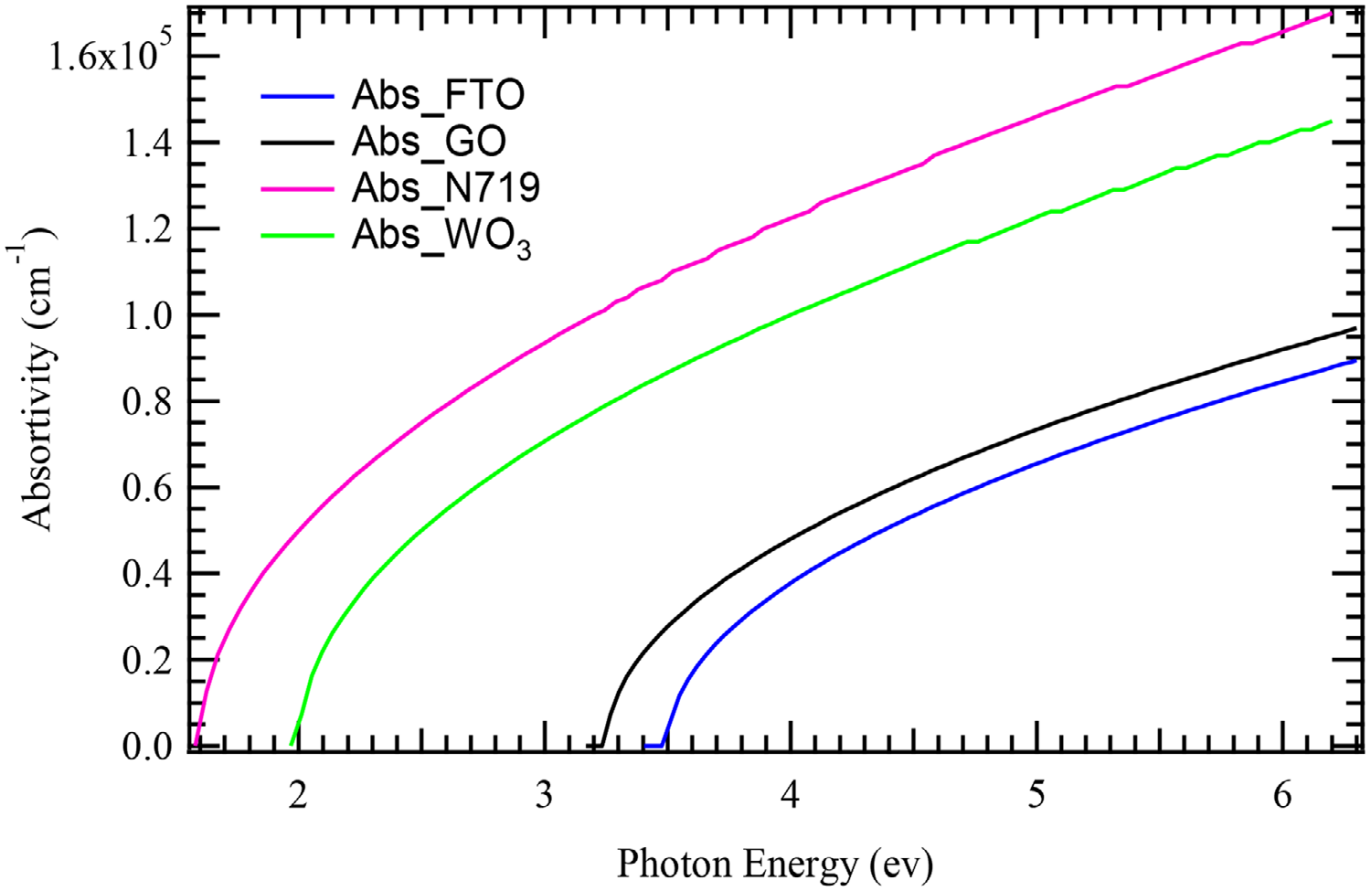
Absorption profiles of the cell layers.

The absorption characteristics of the materials in **Figure** **3** play a critical role in determining their suitability for photovoltaic applications. The low absorption exhibited by FTO is advantageous for its role as a transparent conducting oxide, thereby maximizing photocurrent generation. In contrast, GO shows absorption behavior that suggests its potential contribution to charge transport, which may help in reducing recombination losses and improving overall device efficiency. The dye sensitizer, N719, demonstrates strong absorption across a broad wavelength range, indicating its high efficiency in capturing light and generating excitons. This characteristic is particularly beneficial in ssDSSCs, where effective light absorption directly correlates with enhanced photocurrent production. WO_3_, on the other hand, exhibits notable absorption, which could be useful in facilitating charge transport or serving as an electron extraction layer. Additionally, its absorption properties suggest potential applications in tandem solar cell configurations where multiple layers contribute to enhanced energy conversion.

### 3.3 Performance Metrics of the Model Solar Structure with Temperature

Temperature fluctuations have a notable impact on the performance of ssDSSCs. Elevated temperatures often reduce efficiency by accelerating the degradation of dye molecules and electrolytes, which hampers light absorption and charge generation.^[^ ^20^ ^]^ Additionally, higher temperatures increase electron‐hole recombination rates, further lowering power conversion efficiency.^[^ ^80^ ^]^ On the other hand, lower temperatures can decrease the ionic conductivity of the electrolyte, impairing charge transport and overall cell performance.^[^ ^20^, ^81^ ^]^ Therefore, an optimal temperature range is essential for achieving peak efficiency and ensuring the durability of ssDSSCs.

**Figure 4:**
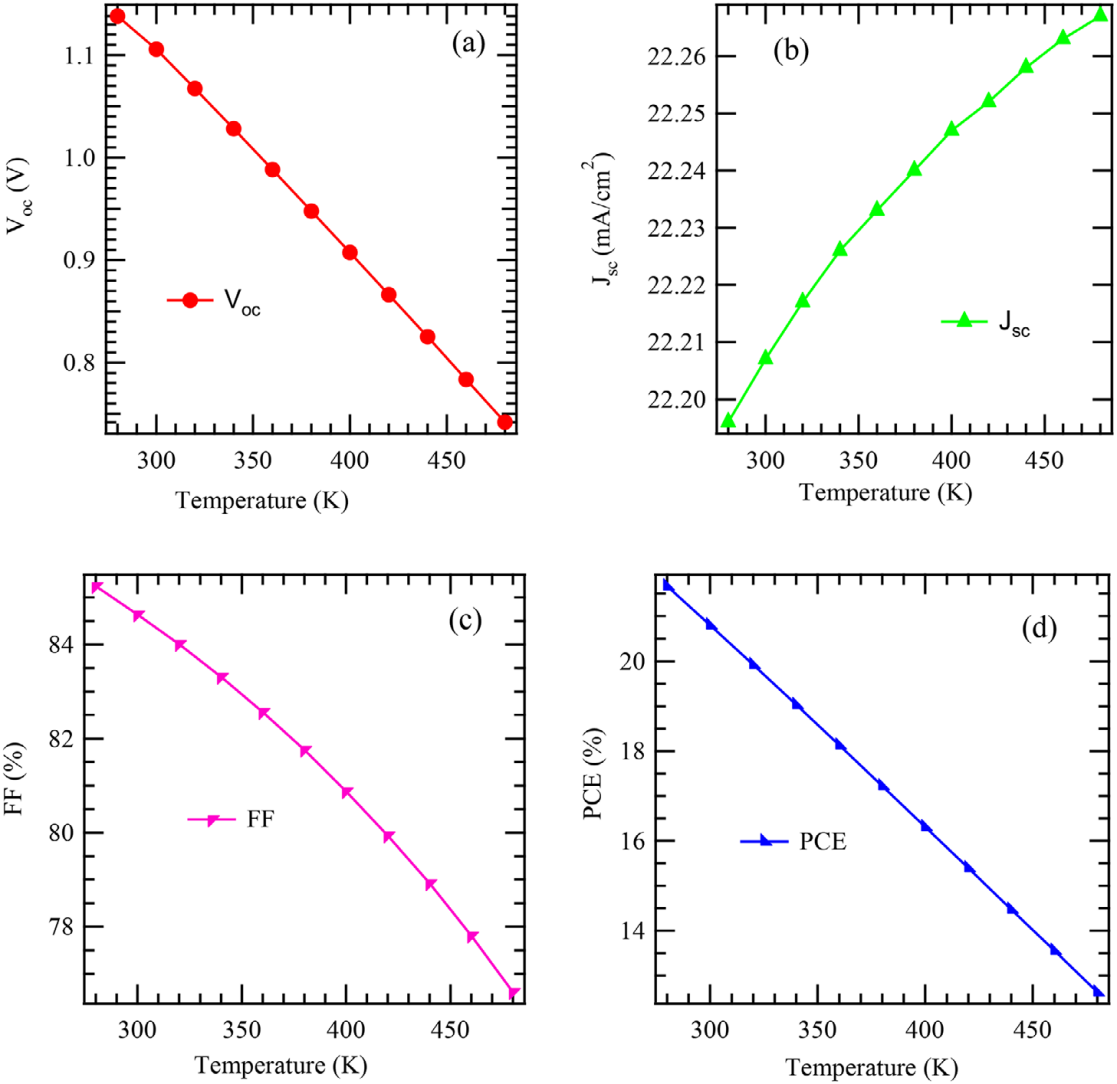
Effect of temperature of electrical parameters.

**Figure** **4**a illustrates the impact of temperature on the V_oc_ of the solar cell device. The plot shows a nearly linear decrease in V_oc_ as the temperature irises from 280 K to 480 K. This inverse relationship between temperature and V_oc_ is a well‐established behavior in photovoltaic devices and can be attributed to several interrelated physical phenomena.^[^ ^82^ ^]^ As temperature rises, the intrinsic carrier concentration within the absorber material increases due to enhanced thermal excitation.^[^ ^58^, ^83^ ^]^ This leads to an exponential increase in the reverse saturation current (I_0_). Further, higher temperatures intensify carrier recombination within the solar cell, particularly non‐radiative recombination, which further reduces the quasi‐Fermi level splitting.^[^ ^84^ ^]^ This results in a lower V_oc_. Another contributing factor is the narrowing of the semiconductor bandgap at elevated temperatures, which limits the maximum possible photovoltage the device can generate. Overall, the trend observed in Figure 3a underscores the temperature sensitivity of the open‐circuit voltage. This degradation in V_oc_ with rising temperature poses a critical challenge for maintaining high performance and efficiency in solar cell applications, particularly under outdoor or high‐irradiance conditions. Toward this end, the decline in V_oc_ with increase in operating temperature is ascribed to uptick in the reverse saturation current (J_0_). Further, the increase in thermal energy results in high‐energy electrons which are more likely to recombine with holes at a higher rate. From Equation 3,^[^ ^85^ ^]^ V_oc_ depends on the J_0._

(3) $$V_{oc}=\frac{KT}{q}\left(\frac{J_{sc}}{J_{0}}+1\right)$$

where an increase in the temperature T decreases *V*_0*c*_ because it is dependent on inverse saturation current density, *J*_0_. K is the Boltzmann constant, while q is the electronic charge. Thus, to increase voltage at the open circuit, it is necessary to decrease*J*_0_. Figure 4b shows the effect of operating temperature on J_sc_ and the effect of temperature variation on FF as shown by Figure 4c.

The trend illustrated in Figure 4b demonstrates a clear and progressive increase in J_sc_ as temperature rises. Initially, at ≈280 K, J_sc_ is recorded at ≈22.22 mA cm^−^^2^. As the temperature continues to rise, J_sc_ steadily climbs, eventually reaching about 22.28 mA cm^−^^2^ at 470 K. The pattern of increase appears to occur in discrete increments, suggesting that the data may have been collected at specific temperature intervals, resulting in a step‐like or piecewise linear representation. This behavior aligns well with the established understanding of temperature effects on semiconductor operational performance, particularly in solar device. Generally, J_sc_ exhibits a positive temperature coefficient, meaning it tends to increase with temperature. This is primarily due to enhanced thermal generation of charge carriers. As temperature rises, the thermal energy provided to the semiconductor increases, thereby promoting more electrons from the valence band to the conduction band.^[^ ^86^ ^]^ This leads to a higher generation rate of electron‐hole pairs, which directly contributes to an increase in current under short‐circuit conditions.

Further, increasing temperature causes a slight narrowing of the band gap in most semiconductors. A narrower band gap reduces the energy threshold required for photoexcitation, enabling a larger proportion of incoming photons even those with relatively lower energy to contribute to charge carrier generation. This, in turn, boosts the overall photocurrent. Moreover, temperature can influence carrier mobility. In some materials, a modest temperature increase may initially improve mobility due to the reduction in carrier trapping. However, at elevated temperatures, increased phonon scattering typically begins to dominate, which can reduce mobility.^[^ ^87^ ^]^ Nevertheless, over the temperature range shown in Figure 3b, the positive impact of increased carrier generation on J_sc_ appears to outweigh any mobility‐related losses. The increasing trend of J_sc_ with temperature observed in Figure 3b is consistent with semiconductor physics. The primary driving factors are the enhanced generation of electron‐hole pairs and the narrowing of the band gap, both of which contribute to a higher short‐circuit current as the temperature rises.

Figure 4c illustrates the variation of FF, as a function of temperature (K) for the proposed solar cell. The graph reveals an inverse relationship between FF and temperature. As the temperature increases, the FF steadily decreases. For instance, at 280 K, the FF is ≈85% but as the temperature rises to 470 K, the FF drops to about 77%. This behavior is represented by a relatively linear downward slope, indicating a consistent decline in FF with rising temperature. This decline has important implications for solar cell performance. The FF is a key indicator of the quality and efficiency of a solar cell, as it reflects the efficiency of the model cell device in converting photon energy to useful electrical power. Several physical mechanisms contribute to the observed decrease in FF with temperature: One of the primary factors driving the decrease in FF is the temperature‐induced reduction in V_oc_. As temperature increases, intrinsic carrier concentration in the semiconductor rises, which in turn elevates the dark saturation current.^[^ ^88^ ^]^

Figure 4d illustrates the influence of temperature on the PCE of the proposed solar cell, revealing a distinct and consistent downward trend as temperature rises. Specifically, the PCE begins at ≈21.67% at 285 K and steadily declines to about 12.5% at ≈475 K, displaying a nearly linear negative correlation between temperature and efficiency. This behavior aligns with what is usually observed in semiconductor‐based photovoltaic devices and can be attributed to several interrelated physical phenomena. First, elevated temperatures introduce additional thermal energy into the semiconductor, which enhances the rate of electron‐hole recombination.^[^ ^57^ ^]^ This recombination reduces the number of charge carriers available for extraction, thereby lowering the current output. Second, an increase in temperature typically results in a narrowing of the semiconductor bandgap, which subsequently reduces the built‐in electric potential.^[^ ^89^ ^]^ This manifests as a decline in the V_oc_, a key determinant of overall efficiency. Because PCE is directly related to V_oc_, any reduction in voltage contributes significantly to performance degradation. Moreover, temperature rise leads to an exponential increase in the reverse saturation current, or dark current, within the solar cell.^[^ ^20^ ^]^ This increased dark current further diminishes the net photocurrent, compounding the losses in overall efficiency. Although carrier mobility may initially benefit slightly from increased temperature, the negative effects of recombination and V_oc_ reduction overwhelmingly dominate, resulting in a net decline in performance.

The temperature range of 280 K to 480 K was selected to evaluate the performance metrics of the solar cell structures because it encompasses both typical and extreme operating conditions. Under normal environmental settings, especially in ambient outdoor applications, solar cells operate within a moderate temperature window, usually between 280 K and ≈350 K.^[^ ^80^ ^]^ However, under intense solar irradiation, poor heat dissipation, or in concentrated photovoltaic systems, the temperature can rise significantly, reaching values as high as 500 K.^[^ ^90^ ^]^ Therefore, this extended range enables a comprehensive assessment of the solar cell's behavior across practical and stress‐induced scenarios. Toward this end, understanding how key parameters evolve with temperature is essential for optimizing the device for specific applications, including solar energy harvesting in hot climates, building‐integrated photovoltaics (BIPV), or space environments where thermal management is challenging.^[^ ^91^ ^]^

### 3.4 Impact of QE and J‐V Characteristics at Varying Shunt Resistance and Series Resistance

The operational characteristics of ssDSSCs are significantly modulated by internal resistive losses, primarily attributed to Rs and Rsh.^[^ ^20^ ^]^ The efficiency of the solar cell in converting light to electricity hinges on these resistances, which impact its QE a measure of light capture and conversion at different wavelengths and its J‐V characteristics, illustrating electrical behavior under various conditions.^[^ ^92^ ^]^ Ideally, a high QE across the solar spectrum and a J‐V curve that approximates a rectangular shape are desired, as these indicate efficient power delivery. However, internal resistances can compromise this ideal performance.

Series resistance acts as an impediment to the flow of electrical current within the solar cell.^[^ ^93^ ^]^ This resistance can stem from factors such as suboptimal electrical contacts or the intrinsic resistance of the materials used in the cell's construction. When series resistance is high, it restricts the flow of current, particularly under conditions of high current demand.^[^ ^94^ ^]^ This results in a J‐V curve that deviates from the ideal rectangular shape, exhibiting a reduced fill factor and a lower J_sc_. As a consequence, the overall PCE of the cell is diminished. Although the primary impact of series resistance is on the J‐V characteristics, it can also indirectly reduce the amount of charge that is extracted from the device, thereby affecting the measured QE. Shunt resistance, in contrast, represents the leakage pathways through which current can bypass the intended circuit.^[^ ^95^ ^]^ These pathways can arise from defects in the cell's materials or imperfections at the interfaces between different layers. A low shunt resistance allows current to leak out of the cell, resulting in a decrease in the V_oc_ and the FF.^[^ ^96^ ^]^ This effect is particularly pronounced under low light conditions. Similar to series resistance, the primary impact of shunt resistance is on the J‐V curve. However, if the shunt resistance is sufficiently low, it can result in the loss of generated charge carriers, thereby indirectly lowering the effective QE of the device.

In the context of ssDSSCs, the complex structure of these cells renders them particularly susceptible to variations in both series and shunt resistance.^[^ ^20^ ^]^ Consequently, meticulous control over the materials and fabrication processes is essential to minimize these resistances and optimize cell performance. In summary, the pursuit of high‐performance ssDSSCs necessitates the minimization of series resistance to facilitate efficient current flow and the maximization of shunt resistance to prevent current leakage. By optimizing these parameters, researchers can enhance both the QE and J‐V characteristics of ssDSSCs, thereby advancing the efficiency of solar energy conversion.

**Figure 5:**
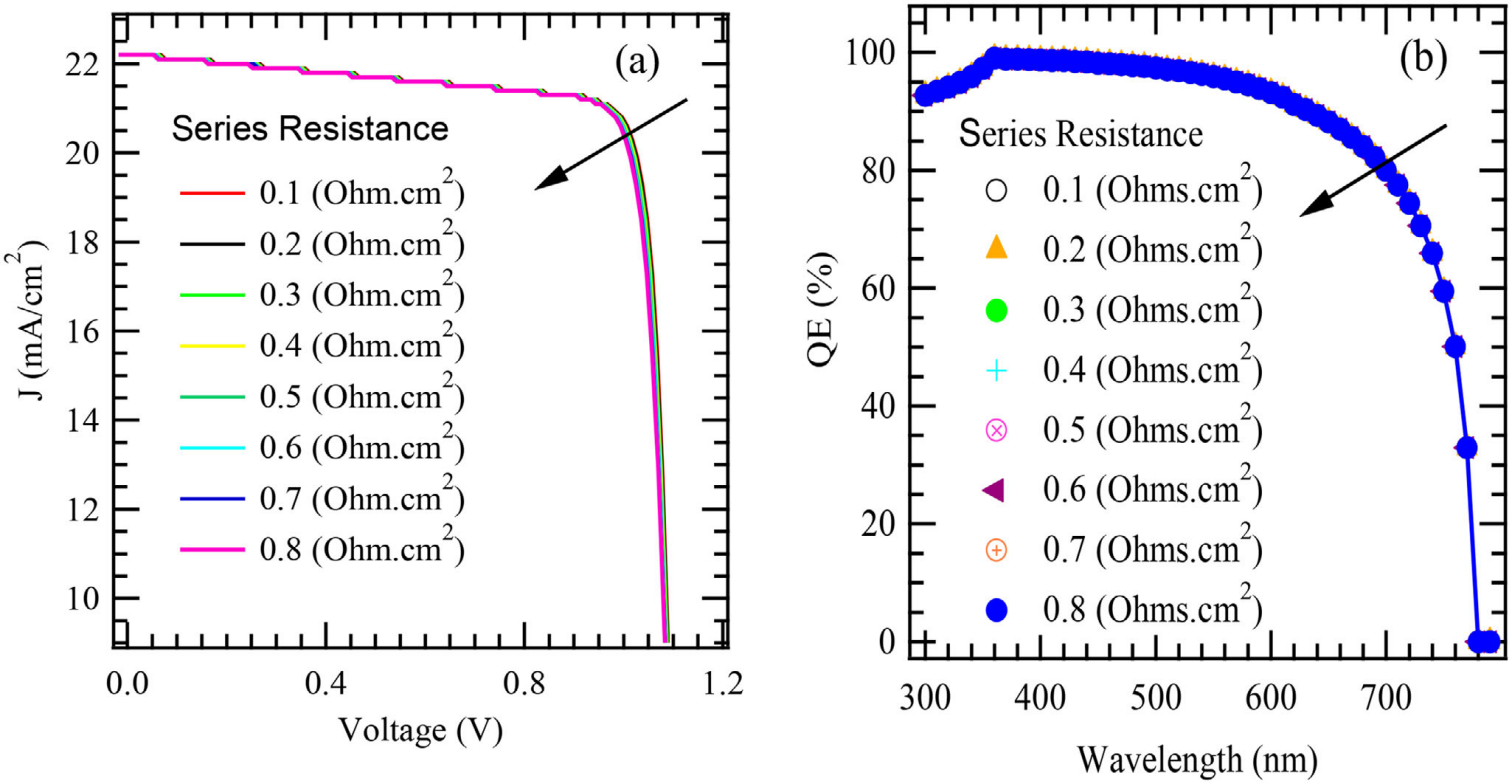
The influence of series resistance on the a) J‐V and b) QE characteristics.

**Figure** **5**a presents the impact of Rs on J–V characteristics of the model device. A series of J–V curves corresponding to different Rs values, ranging from 0.1 to 0.8 Ω cm^2^, are displayed to reveal how varying resistance influences the cell's performance. As the series resistance increases, the J–V plot deviates progressively from ideal behavior, particularly near the maximum power point^[^ ^97^ ^]^ commonly referred to as the “knee” of the curve. This deviation manifests as a noticeable droop or softening in the forward bias region, which leads to a decrease in the performance metrics of the cell device.

The most pronounced consequence of increased Rs is its detrimental effect on the maximum power output (P_max_). Since P_max_ is the product of current and voltage at the knee of the curve, any increase in internal resistance introduces an additional voltage drop (I × Rs), effectively shifting the operational point to lower voltages and/or current densities.^[^ ^98^ ^]^ This results in a visibly smoothed or flattened “knee,” which signifies a decrease in both power density and conversion efficiency. Overall, the trends in Figure 4a underscore that while series resistance has negligible influence on J_sc_ and V_oc_ within the studied range, it significantly deteriorates the fill factor and thus the overall power output. These findings emphasize the critical need to minimize series resistance in the design and fabrication of high‐performance solar cells.

Figure 5b presents the variation of QE as a function of wavelength for different values of series resistance in the model cell architecture. Each curve in the plot corresponds to a distinct series resistance value, ranging from 0.1 to 0.8 Ω cm^2^. It is observed that lower series resistance leads to higher QE across the entire spectral range, with the most pronounced performance seen at 0.1 Ω cm^2^. At this resistance level, the solar cell achieves a near‐maximum QE approaching 100%, particularly within the visible spectrum range,^[^ ^99^ ^]^ indicating highly efficient photon‐to‐charge‐carrier conversion. As series resistance increases; however, a progressive degradation in QE becomes evident especially at longer wavelengths (600–900 nm). For higher resistance values such as 0.6 and 0.8 Ω cm^2^, the QE curves begin to drop off much faster, ≈550–600 nm, and approach zero at ≈780 nm, highlighting the adverse effect of series resistance on the collection of charge carriers generated at lower photon energy. This is likely due to the fact that these photons penetrate deeper into the absorber layer, where carrier collection is more sensitive to resistive and recombination losses. Higher series resistance causes greater internal voltage drops, reducing the terminal output voltage of the cell.^[^ ^100^ ^]^ It also lowers the FF and the overall PCE which may be attributed to increased resistive power losses. In some cases, it may also influence internal electric fields or promote recombination, particularly under high illumination conditions. From the Q‐E characteristic curve (Figure 5b), identifying noticeable differences due to variation in series resistance is difficult, implying that there is no marked effect in QE with change in series resistance.

**Figure 6:**
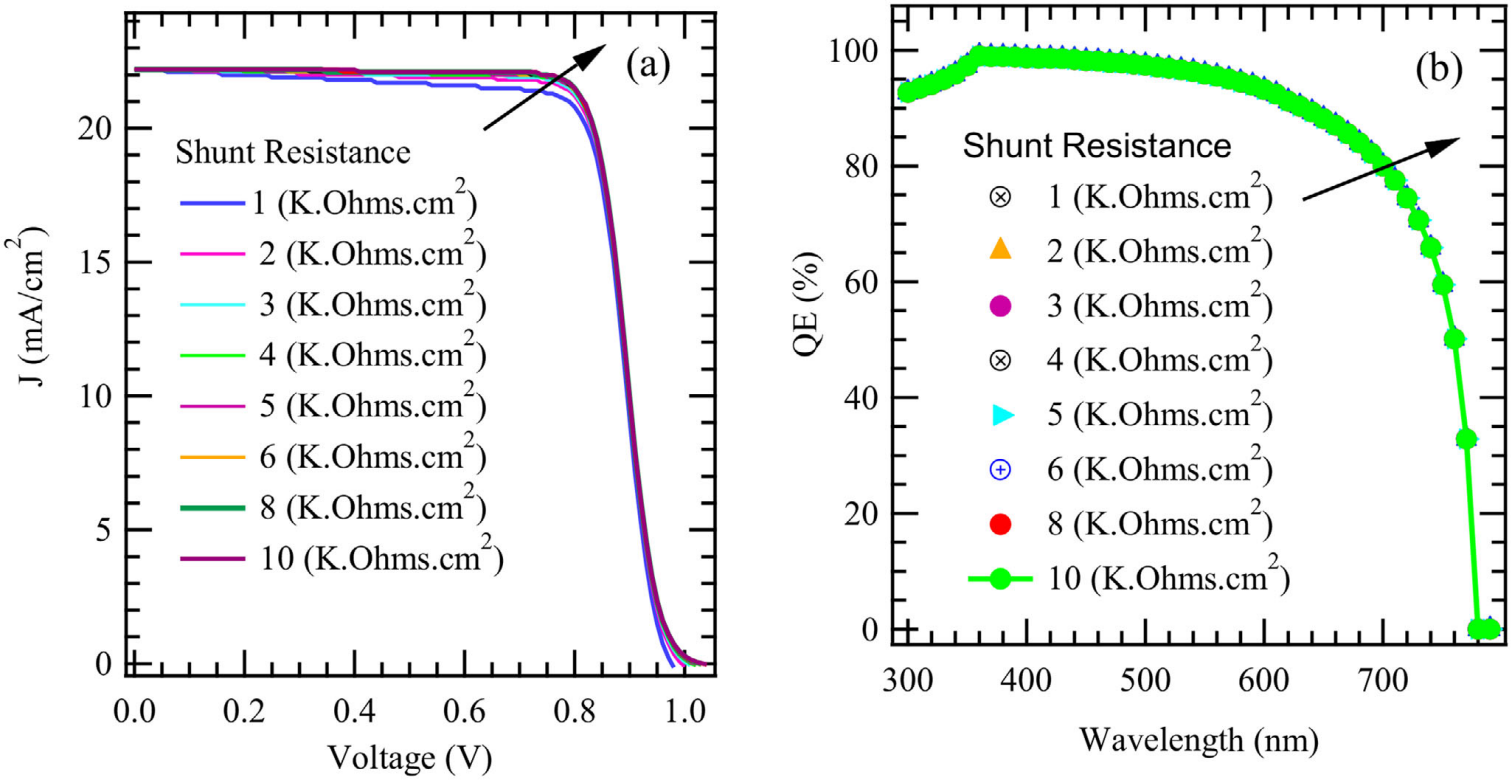
The influence of shunt resistance on J‐V and QE characteristics.

**Figure** **6**a presents the effect of varying Rsh on the current density characteristics of the model solar cell. This demonstrates how different values of Rsh, ranging from 1 to 10 kΩ cm^2^, influence the device performance. At lower values of shunt resistance, such as 1 to 3 kΩ cm^2^, the J–V curves exhibit significant deviations from ideal diode characteristics. As the shunt resistance increases beyond 5 kΩ cm^2^, the J–V curves progressively approach the ideal shape, characterized by a steeper rise near the short‐circuit condition and a more pronounced plateau. This indicates that the leakage current is significantly minimized, allowing the cell to retain more of the photogenerated current. Notably, at higher Rsh, such as 8 to 10 kΩ cm^2^, the improvements reach a plateau, so that further increases in shunt resistance does not impact the performance metrics of the cell significantly. Nonetheless, a high shunt resistance remains a desirable trait in solar cell design, as it ensures minimal parasitic losses and efficient carrier collection. Figure 5a clearly illustrates that increasing the shunt resistance has a beneficial effect on the J–V characteristics of the solar cell. Therefore, high Rsh values contribute to reduced leakage currents, improved fill factors, and enhanced overall efficiency. This is consistent with Equation 4,^[^ ^101^ ^]^ which shows decrease in the J_sc_.

(4) $$I=I_{ph}\left[\exp\frac{q\left(V+IR_{s\;}\right)}{nkT}-1\right]-\frac{V+IR_{s}}{R_{sh}}$$

Here, I*, q, I_ph_, n, k, T, R_sh_, and R_s_* is load current, the electron charge, light‐generated current, ideality factor, Boltzmann's constant, shunt resistance, and series resistance, respectively.

Figure 6b presents the influence of varying R_sh_ on the QE characteristics of the cell architecture. As depicted, the QE response changes significantly with increasing shunt resistance, ranging from 1 to 10 kΩ cm^2^. At lower shunt resistance values specifically 1 to 3 kΩ cm^2^ the QE is markedly suppressed across most of the spectral range. This reduction in quantum efficiency can be attributed to the presence of significant leakage current pathways that allow photo‐generated charge carriers to bypass the external load. Such parasitic pathways reduce the number of carriers contributing to the photocurrent, thereby lowering the QE.

As the shunt resistance increases to intermediate values (≈4 to 6 kΩ cm^2^), there is a noticeable improvement in the QE spectra. This improvement indicates a reduction in leakage losses and enhanced carrier collection, although the QE still does not reach its optimal potential. When the shunt resistance is further increased to higher values such as 8 and 10 kΩ cm^2^ the QE characteristics become nearly ideal, with a relatively flat and enhanced spectral response across a broad wavelength range. This behavior suggests that high shunt resistance effectively suppresses leakage currents and supports more efficient charge separation and collection, resulting in better device performance.

## 4 Impedance Spectroscopy Analysis

The AC electrical behavior was studied by measuring impedance over a frequency range of 1 kHz to 1 MHz and a temperature range of 280 K to 350 K. Software tools, Z‐View and Origin Lab, were utilized for efficient data management and system control. Analysis of the current's amplitude and phase yielded the complex impedance (Z*). From this, the real and imaginary impedance, dielectric losses, and the imaginary part of the electric modulus (M*) were determined and their frequency dependence at different temperatures was graphically represented.^[^ ^102^ ^]^ In the analysis of electrical properties of semiconductor materials, it is important to apply the term electrical resistance for data processing.^[^ ^103^ ^]^ The well‐known Ohm's Law provides a definition of ohmic resistance (*R* (𝛺)) as the quotient of voltage (V) and current (I), mathematically represented by *R* (𝛺) = 𝑉/𝐼. However, its applicability is restricted to direct current (DC) scenarios. To gain a deeper insight into a system's electrical response under more general conditions, a more encompassing approach is required. This approach, known as the generalized Ohm's Law, utilizes the concept of impedance instead of simple resistance.^[^ ^104^ ^]^

An alternating current (AC), typically with a sinusoidal form (*I* = (ω𝑡 + ∅), where *I*_𝑚_ is the current maximum amplitude, ω is the angular velocity given ω = 2𝜋𝑓, and ∅ is the respective, phase angle, is applied as a stimulus, and the voltage (*V* = *V*_𝑚_𝑐𝑜(𝑤𝑡 + 𝜃) produced at the system terminals is measured, where *V*𝑚 is the current maximum amplitude, and 𝜃 is the respective phase angle.^[^ ^104^ ^]^ The impedance is then determined by dividing the measured voltage by the used current; if performed in the frequency domain, the impedance Z is given by Z* = (*V*_𝑚_/*I*_𝑚_) ×𝑒 − 𝑗(𝜃 − ∅), or in the complex form, *Z* = *R* + 𝑗*X*, where *R* is the resistance of the system (*Z*′ = *R* = *Z*𝑐𝑜𝑠(𝜃 − ∅)), and *X* is the reactance (*Z*″ = *X* = *Z*𝑠𝑖𝑛(𝜃 − ∅)).^[^ ^104^ ^]^ While alternating current and voltage are in phase in a purely resistive system (such as a single resistor), a comprehensive analysis of electrical system behavior necessitates the inclusion of other components like inductors and capacitors. These, along with other electrical elements are characterized by their inductance (L) and capacitance (C). A key distinction is that resistors dissipate energy as heat, whereas these reactive components store energy in electric and magnetic fields. Impedance is usually measured using a low‐level signal to maintain linear conditions, akin to DC measurements.^[^ ^103^ ^]^ Moreover, studying a material's electrical response across various frequency ranges, referred to as frequency sweeps, is a valuable method, given that the voltage potentials are sinusoidal.^[^ ^105^, ^106^ ^]^

### 4.1 The Effect of Temperature

**Figure 7:**
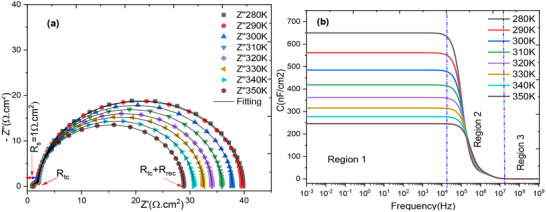
a) Nyquist representation; b) Evolution of cell capacitance as a function of frequency at all temperatures.

To assess the intrinsic properties of the cells, an impedance spectroscopy analysis was performed on the FTO/WO3/N719 Dye/GO/C model cell structure. This section aims to correlate the photovoltaic parameters with the impedance parameters. A simplified equivalent circuit diagram for the dye‐sensitized hybrid solar cell is presented in Figure 20, vide infra. The profile of this plot clearly demonstrates that the Nyquist plot features two semicircles: one at high frequency and one at low frequency. **Figure** **7**a shows the Nyquist diagram, revealing the presence of two distinct resistances: a low‐frequency resistance associated with charge recombination, and a high‐frequency resistance related to charge transport within the cell. Figure 7b illustrates the evolution of the cell capacitance as a function of frequency for all simulated temperatures. This evolution highlights three clearly defined regions:

***Region 1***: a capacitance plateau observed in the frequency range from 1 mHz to 100 kHz;
***Region 2***: a sharp drop in capacitance between 100 kHz and 10 MHz;
***Region 3***: the capacitance reaches a limiting value, denoted CHF, in the frequency range from 10 MHz to 1 GHz.

**Figure 8:**
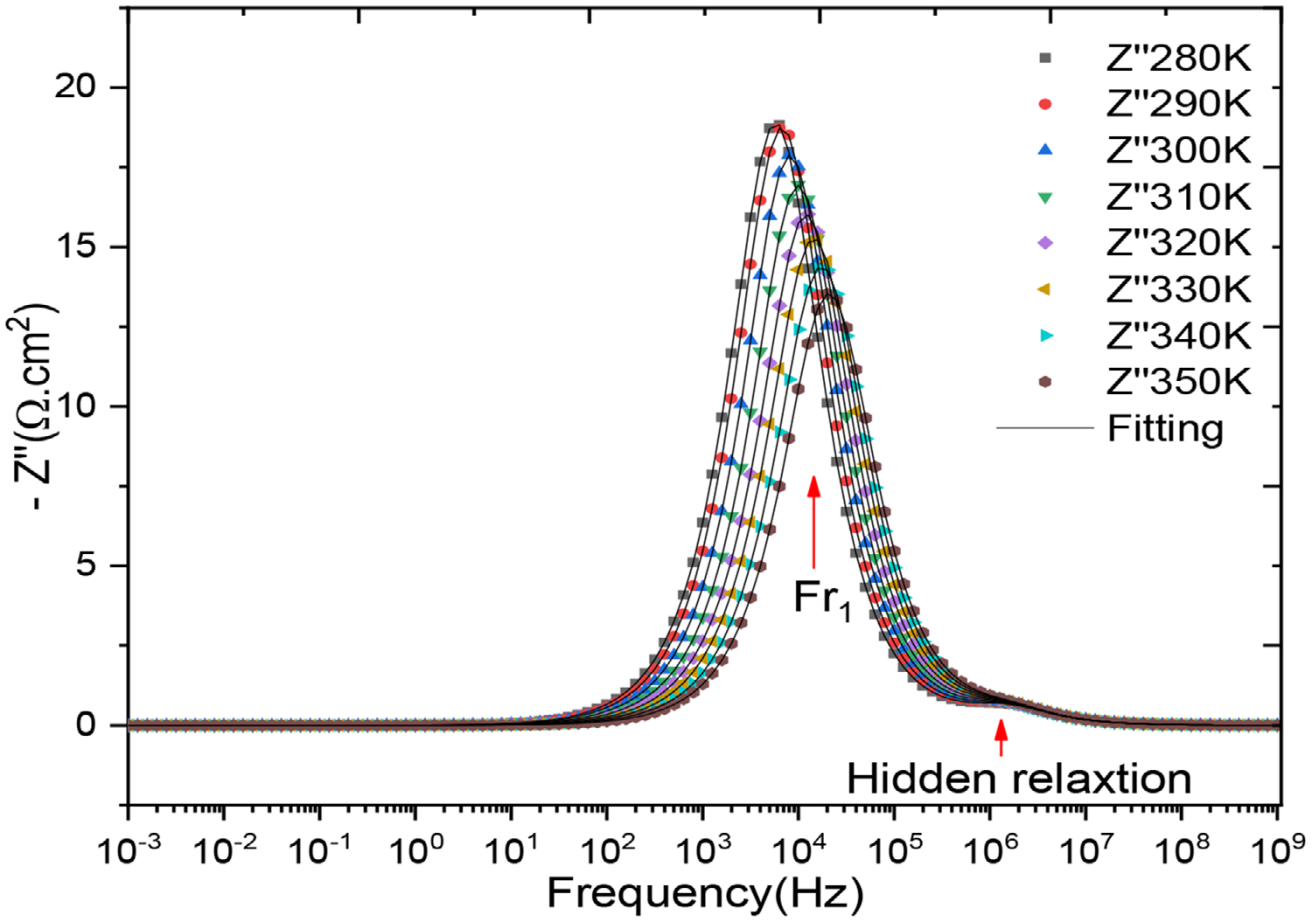
Variation of the imaginary part as a function of frequency for all temperatures.

**Figure** **8** shows how the imaginary part changes with frequency at different simulated temperatures. This plot reveals two distinct relaxations: one at low frequency, associated with the recombination phenomenon, and a masked relaxation at high frequency, related to charge transport through the cell. We observe a greater amplitude of the relaxation frequency ≈10 kHz for the imaginary part. The elevated resistance at this frequency, attributed to relaxation phenomena such as charge recombination, implies substantial electrochemical activity, which is probably a consequence of the incorporated dye.^[^ ^107^ ^]^ At higher frequencies, ≈100 kHz, the amplitude of the response significantly decreases. This lower amplitude is likely to be associated with slower or less pronounced relaxation phenomena, which may be linked to processes such as charge transport through the material or across interfaces. These processes do not produce a substantial variation at this frequency.

The low imaginary part of the complex impedance in the frequency range [1 mHz – 100 Hz] which can have several interpretations. The small imaginary component at low frequencies could mean that capacitive (energy storage) or reactive effects are minimal or absent. This suggests that there is little accumulation or release of energy at these frequencies, potentially due to inefficient charge/discharge processes at low frequencies. We interpret this as a sign that relaxation processes or dynamic phenomena are not strongly occurring at these frequencies. In other words, the system does not exhibit significant reactive behaviors at low frequencies.^[^ ^108^ ^]^

**Figure 9:**
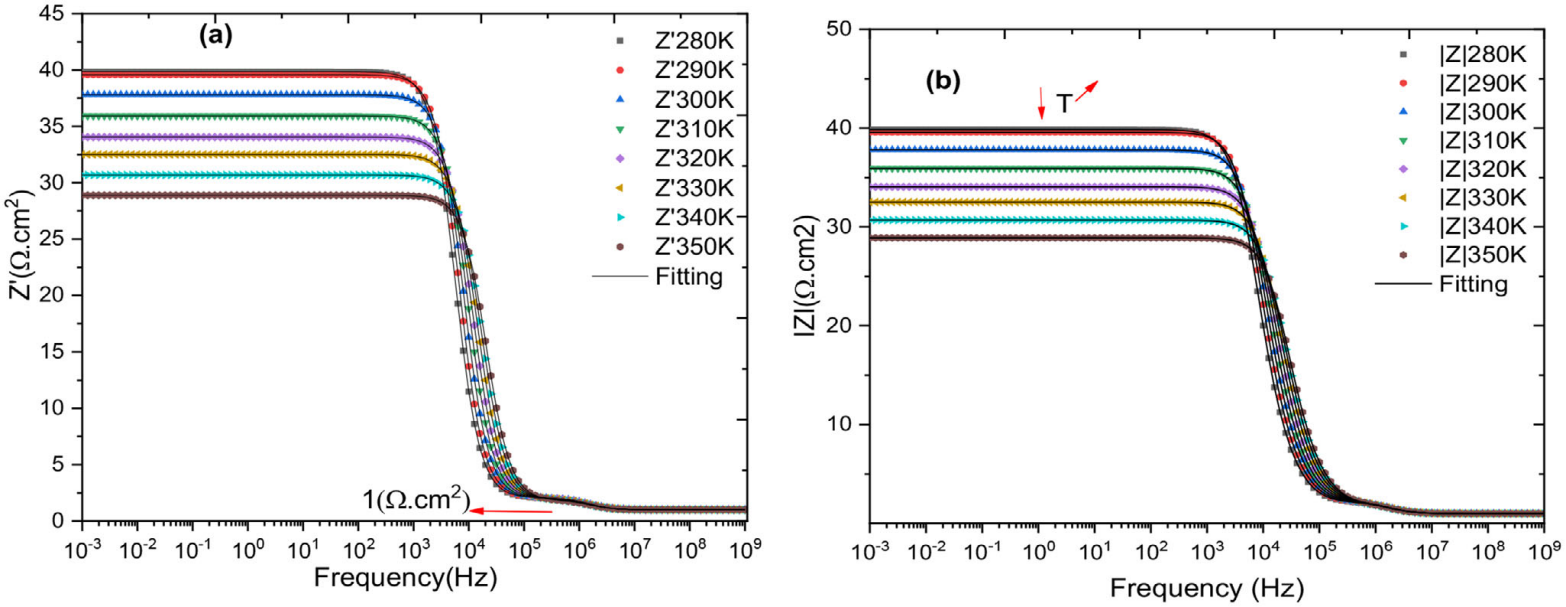
Plot the a) real part and b) the modulus of Z* as functions of frequency, showing the data for all temperatures.

**Figure** **9**a presents the variation of the real part of the impedance (Z′) as a function of frequency for all simulated temperatures. This plot underscores the effect of temperature on the resistive component of the complex impedance, providing insights into the thermal stability of the cell. Figure 9b depicts the frequency‐dependent modulus of the complex impedance (∣Z∣) across the same temperature range. By comparing the trends in Z′ and ∣Z∣, it becomes evident that the imaginary part of the complex impedance (Z′′) exerts a negligible influence on the impedance modulus in the proposed device. This suggests that the cell maintains stable impedance characteristics under thermal variations, which is beneficial for reliable operation.

**Figure 10:**
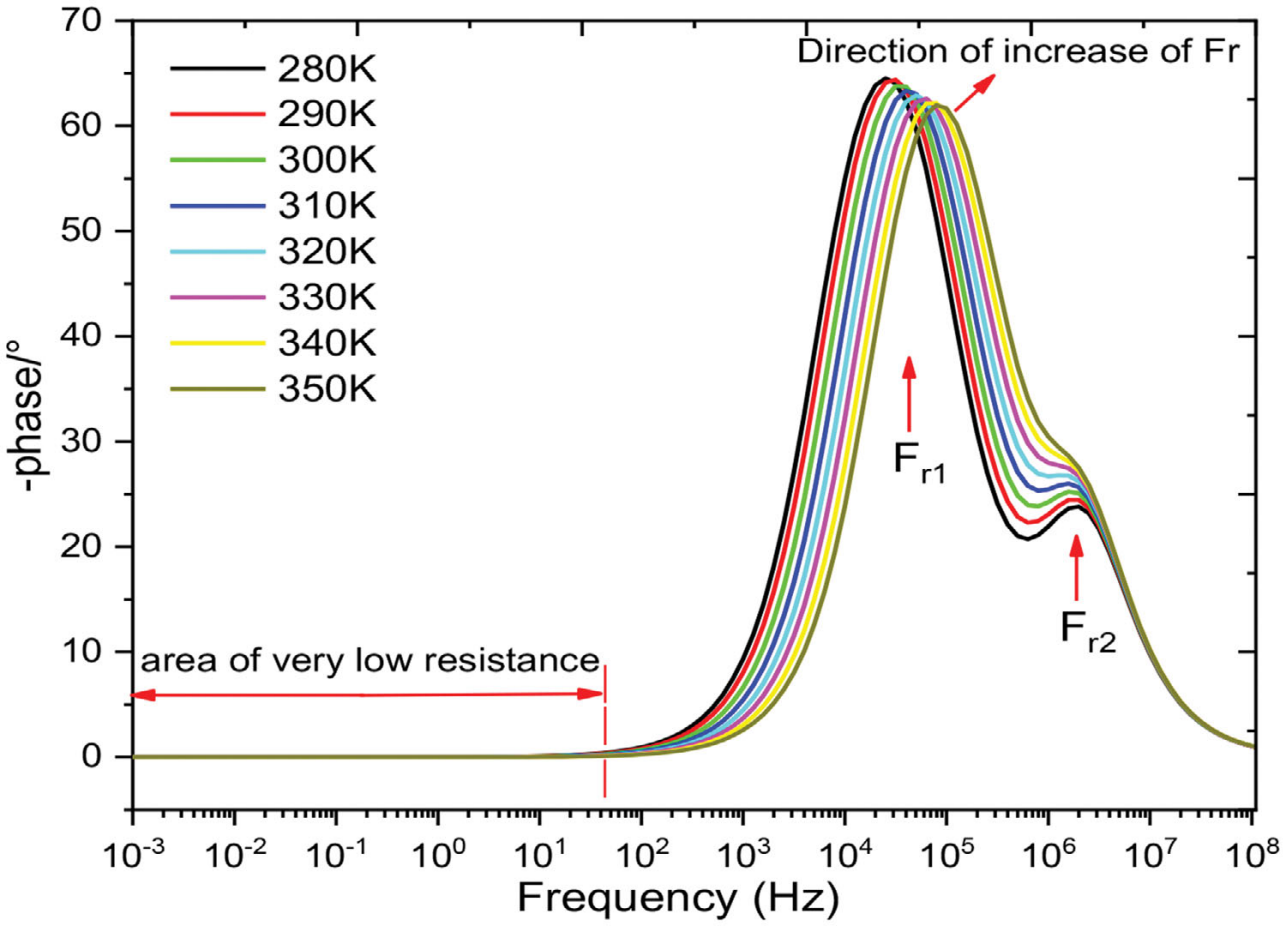
Shows how the phase changes with frequency at different temperatures.

The change in the phase angle with frequency at different temperatures is demonstrated in **Figure** **10**. This representation clearly reveals two distinct relaxations: one with a significant amplitude and the other with a weaker amplitude. The first relaxation is attributed to the recombination phenomenon, while the second is related to charge transport within the cell.^[^ ^36^ ^]^ These findings align with previous studies on the dynamics of charge transport and recombination in similar systems.^[^ ^20^, ^36^ ^]^ We notice that the relaxation frequency shifts to higher frequencies as the temperature increases due to the increased thermal energy available to charge carriers. At higher temperatures, particles or charge carriers (such as electrons or ions) gain more kinetic energy, allowing them to respond more quickly to oscillating electric fields. These results in an increase in the frequency at which these charge carriers can reorganize or move, thus shifting the relaxation frequency to higher values. This phenomenon is often observed in dielectric or conductive materials, where the relaxation process is related to the dynamics of charge transport.

**Figure 11:**
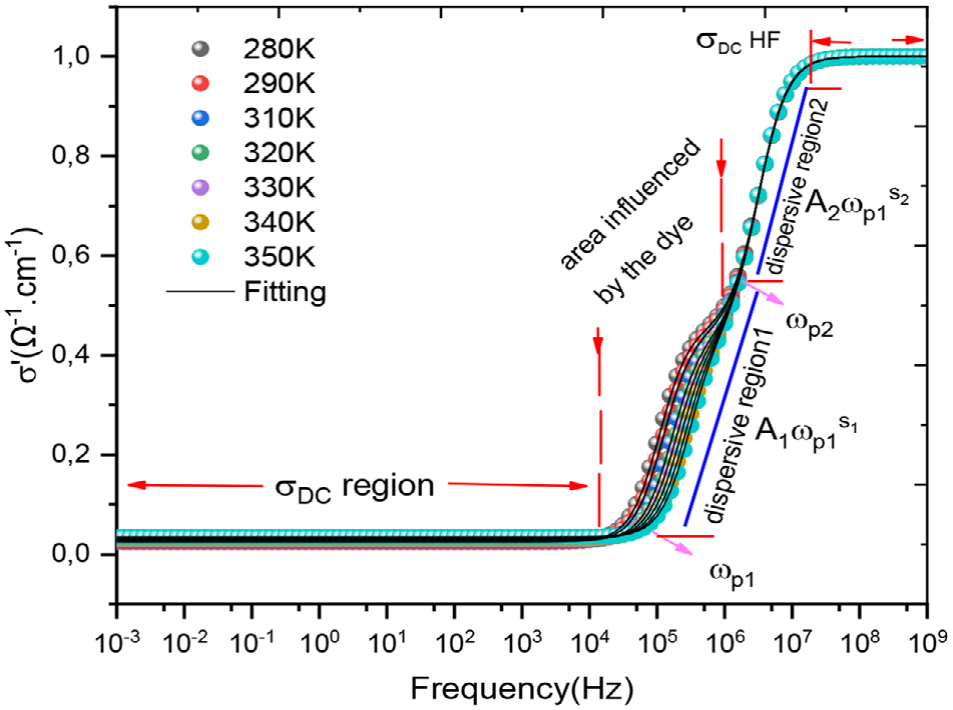
Variation of the real part of the conductivity as a function of frequency for all temperatures.

**Figure** **11** presents the variation of conductivity as a function of frequency for all simulation temperatures. This figure shows the evolution of conductivity across four distinct zones. The first zone, at low frequency in the range [1 mHz–10 kHz], corresponds to direct current conductivity. The second zone displays conduction through two successive jumps, reflecting the jump conduction mechanism described by Jonscher's universal law,^[^ ^109^ ^]^ and the variation of the real part of the electrical conductivity of the cell. The area that shows the complementary jump conduction between the different layers of the cell are well correlated because the coefficients that appear on the angular frequency exponent show the degree of frequency dispersion. The coefficients are very close to each other.

**Figure 12:**
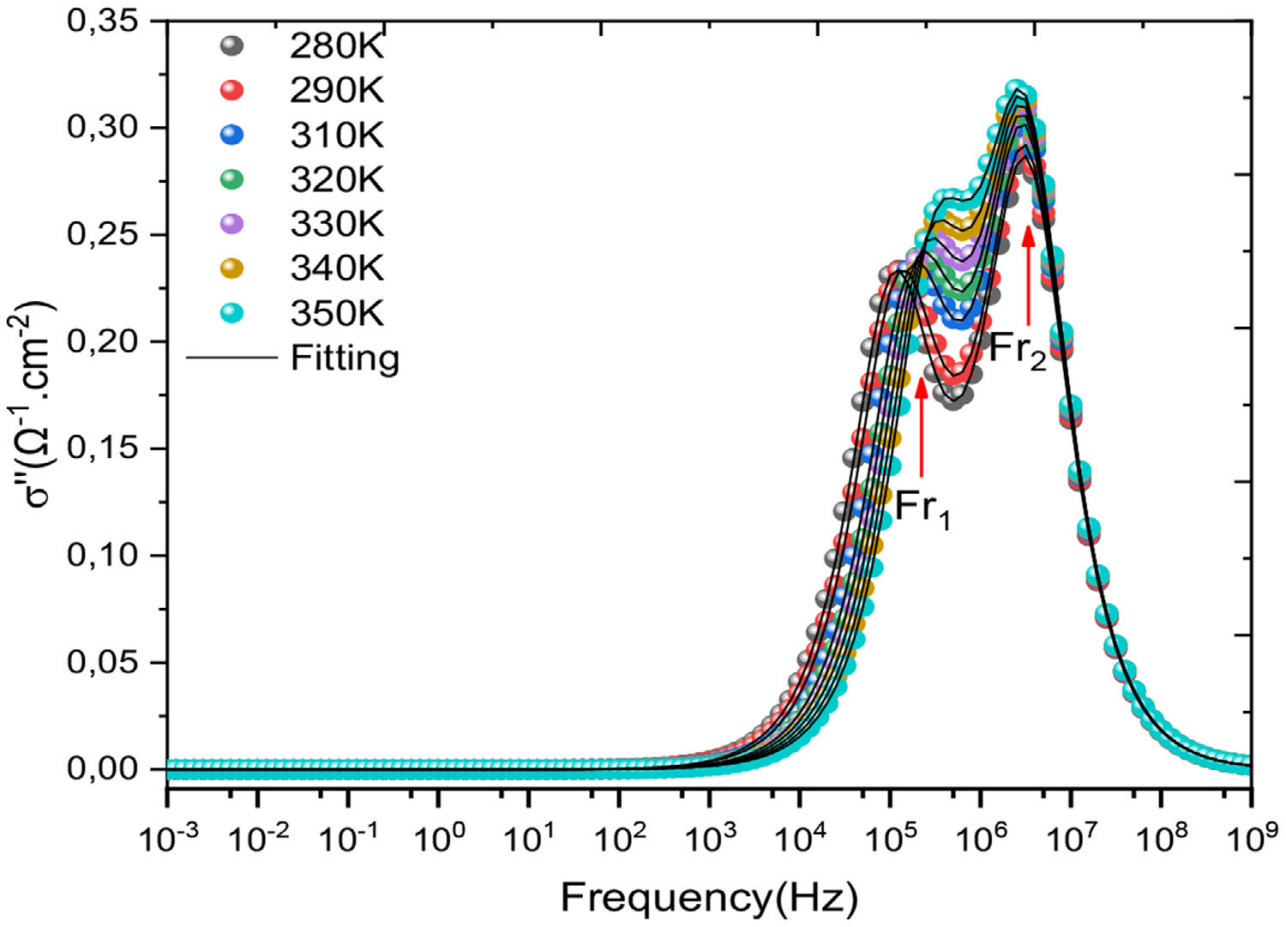
Illustrates how the imaginary component of the complex conductivity changes with frequency across the range of simulated temperatures.

In contrast, **Figure** **12** depicts the frequency dependence of the imaginary part of the complex conductivity for all simulated temperatures.^[^ ^103^ ^]^ This visualization supports the existence of relaxation frequencies, which can be modelled by two equivalent electrical circuit blocks in series with a contact resistance, R_s_. The two relaxations are very close, showing that the combinations of the different layers are well correlated. However, there is a major problem related to the value of the series resistance.

**Figure 13:**
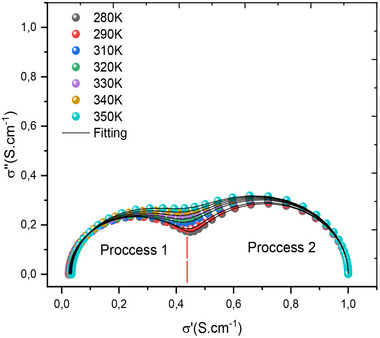
Nyquist plot for complex conductivity as a function of frequency for all different temperatures.

**Figure** **13** shows how the imaginary part of the complex conductivity changes with respect to its real component. From this figure, we clearly show that two relaxations are almost equivalent and present two semicircles in the complex plane of conductivity. It is also noted that temperature makes the regions between the interfaces very active, and hence a good load transporter.

### 4.2 Effect of Series Resistance on Impedance

This section examines the impact of series resistance on cell efficiency and highlights its influence on other hidden factors that are not captured by SCAPS‐1D simulations,^[^ ^74^ ^]^ such as electrothermal losses at the interfaces between different layers and electrode contacts. Impedance spectroscopy, using the Nyquist plot, provides a precise way to assess the effect of series resistance (Rs). Through detailed analysis, we observe that an increase in series resistance negatively affects efficiency. Specifically, as the series resistance increases, the merit coefficient rises, indicating a significant increase in losses.^[^ ^77^, ^78^ ^]^

**Figure 14:**
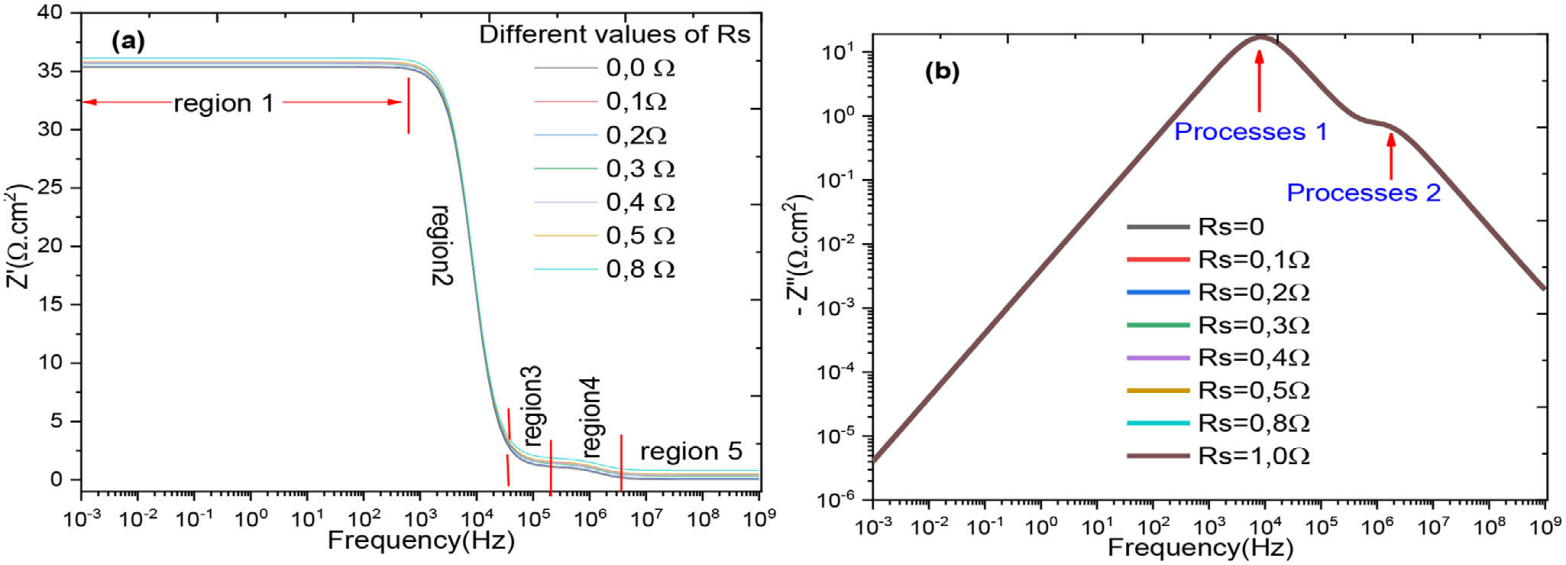
Bode plot for different values ​​of series resistance for a) real impedance and b) imaginary impedance.

**Figure** **14**a,b represents the variation of the real and imaginary parts of the complex impedance, respectively. This representation shows that the real part is dependent on the series resistance for a small variation of the latter. However, the imaginary part is not directly dependent on the series resistance as it simply contributes to the real part of the complex impedance, meaning it adds to the Ohmic contribution of the cell. We observe that the real part of the complex impedance is associated with the resistive components of the system, and in this case, it is directly influenced by the series resistance. This means that small variations in series resistance result in a considerable change in the real component of the impedance. The real part generally reflects the ohmic resistance, which opposes the flow of current, resulting in a decrease in the power supplied by the cell, which reduces its overall operational performance.^[^ ^103^, ^110^ ^]^

The imaginary part of the impedance represents the reactive components of the cell system, such as capacitive or inductive effects. While it is not directly influenced by the series resistance, it still contributes to the overall impedance. In systems with significant capacitive behavior, like electrochemical cells, the imaginary component plays a key role in shaping the frequency response. In contrast, the real part is more directly affected by the series resistance and is responsible for energy dissipation and current flow. Therefore, although the imaginary part does not determine the series resistance, it provides essential information about the system's dynamic behavior. This distinction is crucial in the analysis of electrochemical systems, where understanding both components allows for accurate interpretation of performance and internal processes. The impedance Z of the circuit is defined as the ratio of voltage to current in the case of a sinusoidal signal, and is expressed in ohms. The figure of merit from impedance spectroscopy is calculated as follows:

(5) $$ZT_{impe^{\prime }dance}=\frac{R_{TE}}{R_{ohm}}=\frac{x_{2}-x_{1}}{x_{1}}$$

Here: R_TE_​: total resistance extracted from the Nyquist plot, R_Ohm_​: Ohmic resistance (value at high frequency), x_1_, x_2_​: abscissas of the starting and ending points of the Nyquist arc.

**Figure 15:**
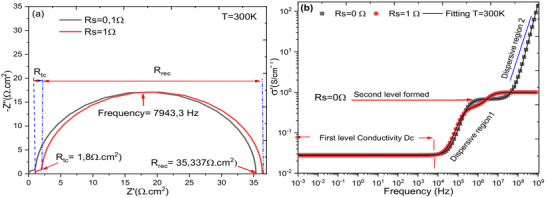
Nyquist diagram for the two values of Rs = 0Ω and Rs = 1Ω.

**Figure** **15**a depicts the simultaneous evolution of the imaginary part as a function of the real part as observed in literature. This representation is made for two values of the series resistance to demonstrate the influence of the series resistance on the Nyquist plot. To determine which configuration is better, we calculate the merit coefficient for the two values of the series resistance. We find zT = 0.949 for Rs = 0Ω, and zT = 0.951 for Rs = 1Ω. Regarding the merit coefficient, it is a good parameter to say that a cell is excellent and has better performance in terms of electrical conductivity according to Equation 6.^[^ ^111^ ^]^ This effect will influence the recombination resistance for charge carriers.

Figure 15b clearly shows the variation in conductivity as a function of two values of series resistance. We found that the series resistance has no influence on DC conductivity over a frequency range from 1 mHz to 10 kHz, covering 40 conductivity points. A remarkable influence is observed between 100 kHz and 10 MHz. This finding is very important for the SCAPS‐1D software.^[^ ^74^ ^]^ It is evident that the frequency range under study is highly efficient. According to this study, we conclude that the frequency range where the cell operates in the active zone is [100 Hz – 10 MHz].

(6) σ=1/ρ=neμ

In this context, n is the number of electrons per unit of volume, e is the fundamental electric charge and µ is electric mobility. This work is based on the research of Nabil Bouri et al.^[^ ^112^ ^]^ to evaluate the charge transfer resistance and the recombination resistance using the Nyquist plot of complex impedance. Further, this plot also allowed us to identify the presence of two relaxation times.

According to the Nyquist diagram, two semicircles can be distinguished: one dominant, extends from low to medium frequency, while the other, located at high frequency, has a very small diameter and is therefore negligible. To analyze the most significant contribution, we used the Nyquist plot applied to the modulus function M∗. In this representation, two well‐defined arcs clearly appear. Moreover, the conductivity versus frequency plot also reveals two characteristic arcs. According to several studies, each semicircle can be modelled, in the ideal case, by an equivalent circuit of the type R//C. However, due to the roughness and heterogeneity of the interfaces formed between the different layers, the capacitor is generally replaced by a constant phase element (CPE).

To understand the conduction mechanism in this cell structure during the intercalation of N719 dye, we observe very significant improvements at the cell interfaces. There are collective activations of electrons which can be interpreted by the periodic jump conduction mechanism, and here, we observe two consecutive jump conduction levels for the series resistance Rs = 0Ω. According to this study and as illustrated in Figure 15, an increase in series resistance leads to non‐periodic conduction energy jumps,^[^ ^113^ ^]^ resulting from the creation of an energetic barrier that enhances the likelihood of recombination.

***Zone 1***: In Figure 15a (Rs = 0Ω, Rs = 1Ω), in this case, the activation energy remains constant and periodic within the frequency range [1 mHz – 10 kHz]. In this zone, the series resistance has no influence. This means that the variation in conductivity is a function of a regular energy profile, with the same energy required for electrons to “hop” from one site to another.

***Zone 2***: In Figure 15a,b, the conductivity likely depends on the frequency in a regular manner, with uniform energy jumps. The conductivity may vary more complexly, with possible energy jumps between the two wells. This could lead to a different response in conductivity at different frequencies, particularly in regimes where electrons can move from one well to another. For Rs = 1Ω, beyond the frequency of 1 MHz, the system introduces more complexity with transitions between two distinct energy wells, leading to more diversified conduction behavior. Therefore, as Rs increases, the system presents the most complex case with multiple levels, resulting in greater variability in conductivity, dependent on multiple energy barriers.^[^ ^112^, ^114^ ^]^

**Figure 16:**
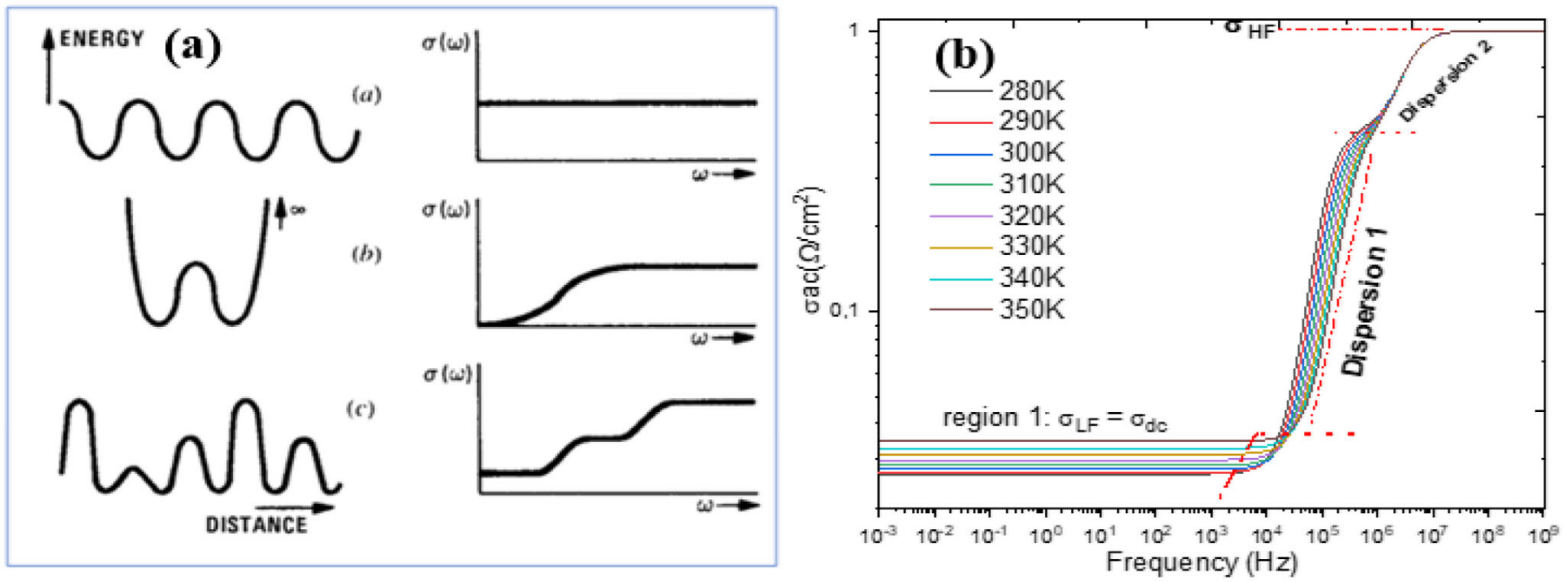
a) Frequency dependence of the hopping conductivity for different potential energy profiles: a) Periodic constant activation energy, b) a single bi‐well, and c) a potential profile with multiple activation energies.^[^ ^103^, ^115^ ^]^ b) Frequency‐dependent electrical conductivity of the perovskite cell at various temperatures.

**Figure** **16**b shows the evolution of electrical conductivity as a function of frequency for all temperatures, in order to confirm the trends observed in Figure 16a. This evolution reveals *four distinct regions* in the behavior of the electrical conductivity.

**Figure 17:**
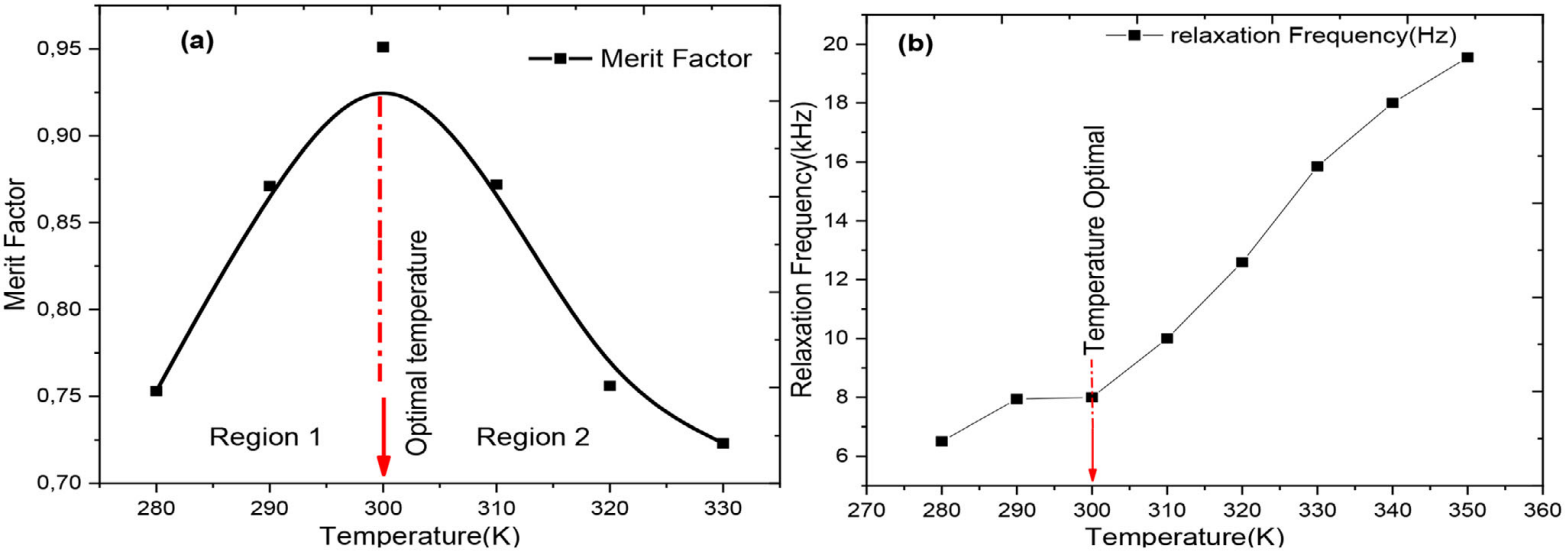
a) The evolution of the figure of merit of the perovskite cell as a function of temperature and (b) variation of the relaxation frequency as a function of temperature.

**Figure** **17**a of merit is a key parameter to study in semiconductors, as it provides valuable information about the thermoelectric within the cell. Figure 17b shows the evolution of the relaxation frequency as a function of frequency for all operating temperatures of the cell, as obtained by simulation.

**Figure 18:**
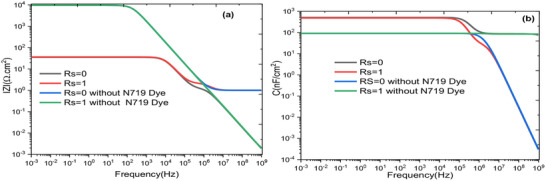
The variation of a) impedance modulus and b) capacitance of the cell as a function of frequency for Rs = 0 Ω and Rs = 1 Ω with and without N719 Dye.

**Figure** **18**a shows the evolution of the overall resistance of the cell with frequency for two values of series resistance, Rs = 0 Ω and Rs = 1 Ω. The N719 Dye active layer has a significant influence on the overall resistance of the cell because, as the cell's resistance increases, the power lost due to Joule heating also increases, leading to a reduction in the cell's power output. It is also noticeable that the presence of the N719 Dye absorber has a considerable effect when the Rs resistance increases.

On the other hand, Figure 18b presents the evolution of the overall capacitance of the device Rs = 0 Ω and Rs = 1 Ω, both with, and without the absorber. It can be seen that the junction capacitance remains constant for Rs = 1 Ω without the absorber. For Rs = 1 Ω and Rs = 0 Ω, the capacitance remains constant at a value of 100 F cm^−^^2^, and then suddenly decreases at a frequency of 1 MHz

For Rs = 0 Ω and Rs = 1 Ω with the N719 Dye absorber, the capacitance was ≈560 F cm^−^^2^ in the frequency range 1 mHz – 100 kHz. However, for Rs = 1 Ω, the capacitance decreases after 100 kHz across the rest of the frequency range without plateauing, whereas for Rs = 0 Ω, the capacitance shows a plateau in the range 1 mHz–100 kHz, then decreases until it reaches a plateau at high frequencies.

### 4.3 Identification of Cell Capacities and Confirmation of Circuits Modelling Impedance Spectra

The complex impedance and modulus modelling of the circuit shown in Figure 22, *vide infra*, follow the same approach as for circuits (a) and (b), although the derivations are significantly more complex. Details regarding the approximations applied can be found in the literature.^[^ ^92^ ^]^ To illustrate the relationships between the real and imaginary components^[^ ^105^ ^]^of both the impedance Z∗ and the modulus M∗ as functions of frequency, the following assumptions are made: R_2_≫R_1_​and C_2_≫C_1_.

The expression for the complex impedance of the model circuit is given by Equation 7:

(7) $$Z*\left(\omega \right)=\frac{R_{1}-j\omega R_{1}^{2}C_{1}}{1+{\left(\omega R_{1}C_{1}\right)}^{2}}+\frac{R_{2}-j\omega R_{2}^{2}C_{2}}{1+{\left(\omega R_{2}C_{2}\right)}^{2}}$$

Considering the limits of frequency for the real part (*Z*’), Equation 8 is deduced.

(8) $$Z^{\prime }\left(\omega \right)=\frac{R_{1}}{1+{\left(\omega R_{1}C_{1}\right)}^{2}}+\frac{R_{2}}{1+{\left(\omega R_{2}C_{2}\right)}^{2}}$$

In the low frequency regime, ω→ 0:

(9) $$Z^{\prime }\left(0\right)=\frac{R_{1}}{1+{\left(0\right)}^{2}}+\frac{R_{2}}{1+{\left(0\right)}^{2}}$$

We find in this limit:

(10) $$Z^{\prime }\left(0\right)=R_{1}+R_{2}$$

At high frequency ω→ ∞:

(11) $$Z^{\prime }\left(\omega \right)=\frac{R_{1}}{1+{\left(\infty \right)}^{2}}+\frac{R_{2}}{1+{\left(\infty \right)}^{2}}$$

Since *C*_2_ ≫ *C*_1_ and *R*_2_ ≫ *R*_1_, the Debye frequency of *R*_2_ *C*_2_ (F_max2_) appears at a lower frequency than the Debye frequency of *R*_1_ *C*_1_ (F_max1_). If we consider the contribution of *R*_1_ *C*_1_ and *R*_2_ *C*_2_ to *Z*’ in the intermediate frequency range between F_max2_ and F_max1_ then we obtain Equation 11.

For bloc 2 (*R*_2_ *C*_2_):

(12) $$Z^{\prime }=\frac{R_{2}}{1+{\left(\omega R_{2}C_{2}\right)}^{2}}$$

At F_max2_, we find this limit: $Z^{\prime }\to \frac{R_{2}}{1+{\left(1\right)}^{2}}\to \frac{R_{2}}{2}$.

If the frequency increases beyond F_max2_, ω→ ∞:$Z^{\prime }\to 0$. Therefore, *R*_1_ *C*_1_ at F_max2_ *ωR*_1_ *C*_1_ will approach zero because it is far from F_max1_, so that for bloc 1(*R*1C_1_) at F_max2_: ω*R*_2_ *C*_2_ = 1,  and the limit: $Z^{\prime }\to R_{1}$ is located.

For higher frequencies approaching F_max1_, we obtain: $\omega R_{1}C_{1}\to 1\Rightarrow Z^{\prime }\to \frac{R_{1}}{2}$

The expression of the modulus function of the circuit is:

(13) $$M*\left(\omega \right)=\frac{1}{C_{1}}.\frac{{\left(\omega R_{1}C_{1}\right)}^{2}+jR_{1}C_{1}\omega }{1+{\left(\omega R_{1}C_{1}\right)}^{2}}+\frac{1}{C_{2}}.\frac{{\left(\omega R_{2}C_{2}\right)}^{2}+jR_{2}C_{2}\omega }{1+{\left(\omega R_{2}C_{2}\right)}^{2}}$$

Toward this end, let us consider the frequency limits of the real part as shown by Equation 14.

(14) $$\left(M^{\prime }\right):M^{\prime }\left(\omega \right)=\frac{1}{C_{1}}\cdot \frac{{\left(\omega R_{1}C_{1}\right)}^{2}}{1+{\left(\omega R_{1}C_{1}\right)}^{2}}+\frac{1}{C_{2}}\cdot \frac{{\left(\omega R_{2}C_{2}\right)}^{2}}{1+{\left(\omega R_{2}C_{2}\right)}^{2}}$$

Low frequency ω→0, the expression of the real part of modulus is: $M^{\prime }\left(\omega \to 0\right)=\frac{1}{C_{1}}.\frac{(\omega \to 0\times {\left(R_{1}C_{1}\right)}^{2}}{1+{\left(\omega \to 0\times R_{1}C_{1}\right)}^{2}}+\frac{1}{C_{2}}.\frac{(\omega \to 0\times {\left(R_{2}C_{2}\right)}^{2}}{1+{\left(\omega \to 0\times R_{2}C_{2}\right)}^{2}}\to \frac{1}{C_{1}}.\frac{0}{1+{\left(0\right)}^{2}}+\frac{1}{C_{2}}.\frac{0}{1+{\left(0\right)}^{2}}=0$. High frequency ω→∞, we obtain Equation 15.

(15) $$M^{\prime }\left(\omega \to \infty \right)=\frac{1}{C_{1}}.\frac{{\left(\infty \right)}^{2}}{1+{\left(\infty \right)}^{2}}+\frac{1}{C_{2}}.\frac{{\left(\infty \right)}^{2}}{1+{\left(\infty \right)}^{2}}\to \frac{1}{C_{1}}+\frac{1}{C_{2}}$$

Since *C*_2_>>*C*_1_ and *R*_2_>>*R*_1_, the Debye frequency of *R*_2_ *C*_2_ (F_max2_) appears at a lower frequency compared to the Debye frequency of *R*_1_ *C*_1_ (F_max1_). Considering the contribution of *R*_1_ *C*_1_ and *R*_2_ *C*_2_ to *M* **′** in the intermediate frequency range between F_max2_ and F_max1_: For *R*_2_ *C*_2_ then we have

(16) $$M^{\prime }=\frac{1}{C_{2}}.\frac{{\left(\omega R_{2}C_{2}\right)}^{2}}{1+{\left(\omega R_{2}C_{2}\right)}^{2}}$$

At F_max2_ : ω*R*_2_ *C*_2_ = 1, we obtain this expression see Figure 17: $M^{\prime }=\frac{1}{2C_{2}}$

For increase in frequency beyond F_max2_, ω→∞, we obtain this limit: $M^{\prime }=\frac{1}{C_{2}}$

Now, considering bloc 1(*R*_1_ *C*_1_), at F_max2,_ *ωR*_1_ *C*_1_ = 1 will tend to zero as we are far from F_max1_ then: For bloc 1(*R*_1_ *C*_1_):

(17) $$M^{\prime }=\frac{1}{C_{1}}.\frac{{\left(\omega R_{1}C_{1}\right)}^{2}}{1+{\left(\omega R_{1}C_{1}\right)}^{2}}$$

At F_max2_, ω*R*_2_ *C*_2_ = 1, $\;M^{\prime }\to 0$

**Figure 19:**
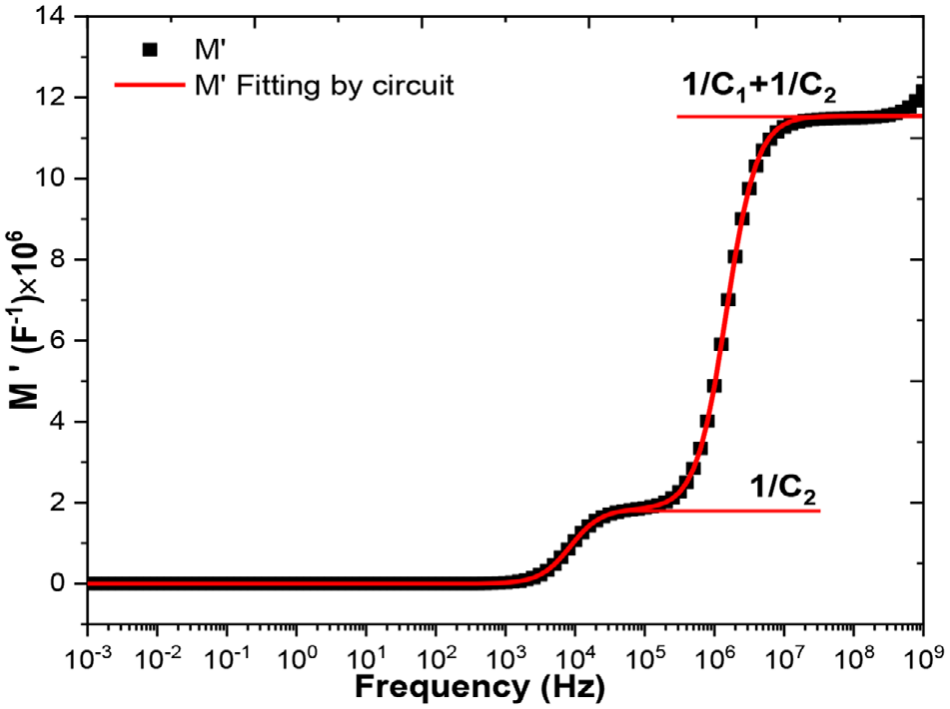
The relationship between M′ as a function of frequency for resistors and capacitors in the model equivalent circuit of the proposed cell structure.

In the high frequency regime toward F_max1_ whereω*R*_1_ *C*_1_ = 1, we obtain the limit:$\;M^{\prime }=\frac{1}{2C_{1}}$. **Figure** **19** depicts the relationship for M′ as a function of frequency superimposed on each other according to the model circuit for the solar cell device. Separating the imaginary part of modulus, we obtain Equation 18:

(18) $$M^{\prime \prime }=\frac{1}{C_{1}}.\frac{R_{1}C_{1}\omega }{1+{\left(\omega R_{1}C_{1}\right)}^{2}}+\frac{1}{C_{2}}.\frac{R_{2}C_{2}\omega }{1+{\left(\omega R_{2}C_{2}\right)}^{2}}$$

**Figure 20:**
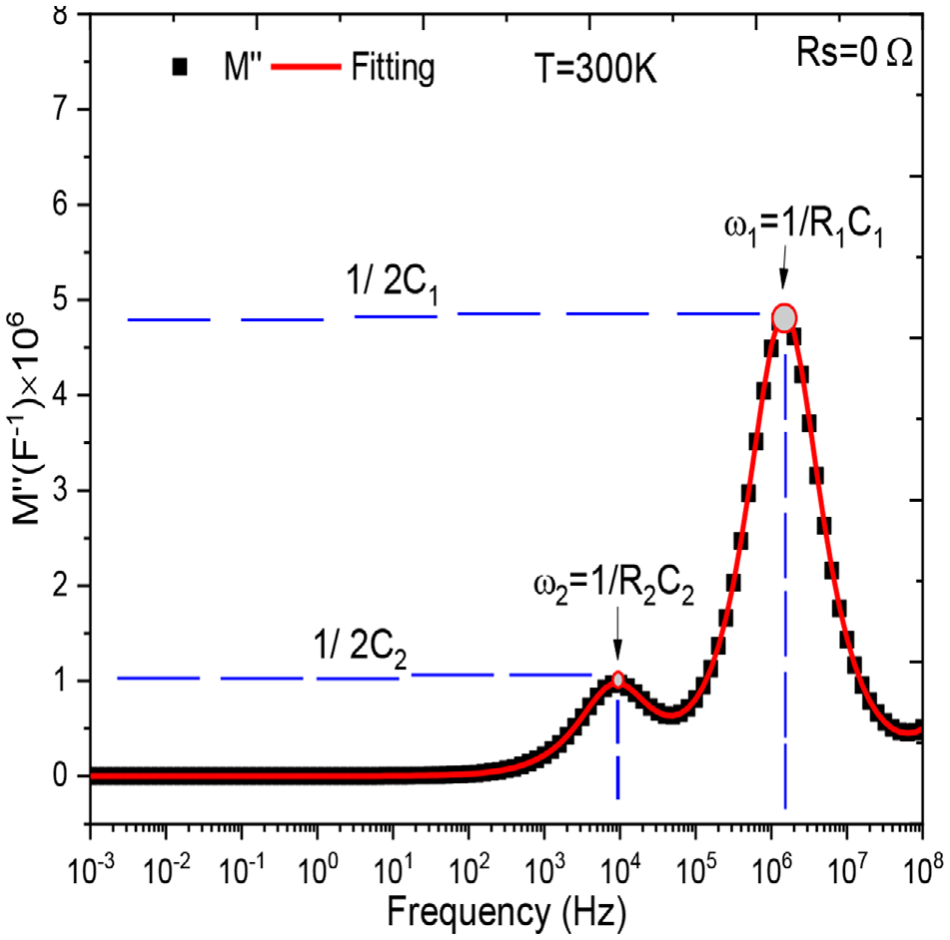
Nyquist diagram for the Modulo M* function.

We see that there are two Debye functions, which will have peaks of values 1/2C_1_ and 1/2C_2_ when the frequency equals F_max1_ and F_max2_, respectively. If the time constants of the two parallel RC circuits are sufficiently different there will be two distinct Debye peaks in the M″ spectroscopic plot (cf. **Figure** **20**).

**Figure 21:**
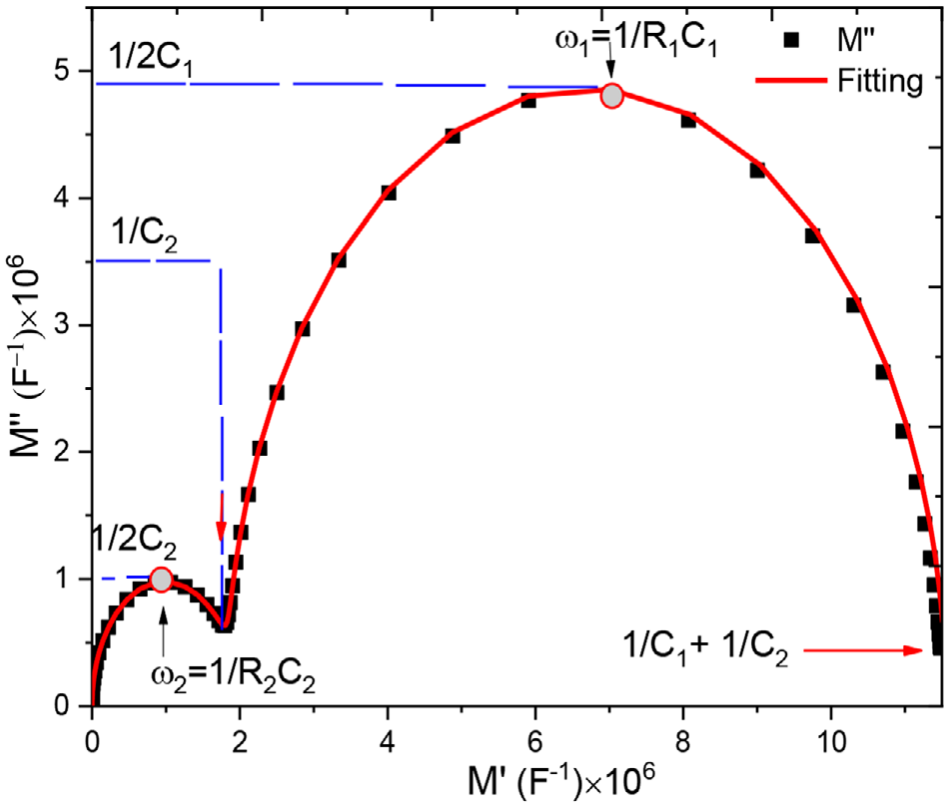
Nyquist plot showing the relationship between M″ as a function of M′ at 300K.

When *M*′ and *M* **″** are combined, the result is a Nyquist plot with two arcs of diameters 1/*C*_1_ and 1/*C*_2_ and corresponding heights of 1/2*C*_1_ and 1/2*C*_2_, respectively. The arc peaks correspond to F_max1_ and F_max2_ (cf. **Figure** **21**). The optimal temperature and photoactive layer thickness were 300 K and 500 nm, respectively, whereas the resistance, Rs = 10 Ohm.

### 4.4 Theoretical Framework for Modelling the Spectra of Modulus (M*) and Complex Impedance (Z*)

Various models of equivalent circuit have been employed for the model device architecture in accordance with the Garcia‐Belmonte series model,^[^ ^116^ ^]^ which facilitates the study of mechanistic processes such as diffusion and recombination, and the model introduced by Mortadi et al (2024),^[^ ^117^ ^]^ comprising three blocks connected in series representing contact and cable effects. In this study, we analyze of the electric modulus (M″) as a function of frequency revealed two distinct peaks at low and high frequencies, indicating the presence of two separate relaxation processes. Further, a peak observed in the imaginary part of impedance (Z″) at low frequency appeared at the same characteristic frequency as one of the M″ peaks, suggesting a shared relaxation response, whereas the second mechanism may be masked. Consequently, the equivalent model circuit illustrated in Figure 21b, *vide infra*, was adopted to examine both the complex impedance (Z*) and electric modulus (M*). The model circuit consists of a series resistance (Rs) connected to two parallel branches, each comprising a resistor and a constant phase element (CPE): (R_1_//CPE_1_); (R_2_// CPE_2_). Each bloc may describe distinct physical phenomena in solar cell structures.^[^ ^117^ ^]^

From Figure 20, the evolution of the imaginary part (M″) as a function of frequency for 0.5 µm thickness at Rs = 0Ω of the photoactive absorber (dye) is chosen as to determine the of the fitting of the proposed equivalent model circuit. Evidently, the best fit was achieved that could to highlight all processes within the circuit. Furthermore, deconvolution procedures were conducted in order to separate the three processes at low, medium, and high frequencies, and also validate the proposed circuit.^[^ ^117^ ^]^ Consequently, further investigation from a theoretical perspective was carried out. The complex impedance of the constant phase element, (CPE‐T, p), is given Equation 19 according to:^[^ ^118^ ^]^

(19) $$Z_{CPE}^{*}\left(\omega \right)=\frac{1}{T{\left(j\omega \right)}^{p}}$$

Here, T represents the pseudo‐capacitance; ω is the angular frequency; *j^2^* = − 1 is the imaginary number, and α is the CPE exponent. For *p* = 1, CPE is a pure capacitor (T = C); for p = 0, CPE is a pure resistance (T = 1/R); and for p = − 1, CPE is an inductor (T = 1/L). The global complex impedance (Z*) of the above circuit can be written as the summation of Rs and the complex impedance (Z*) of each bloc.

(20) $$Z^{*}\left(\omega \right)=Rs+\frac{R_{1}}{1+{\left(j\tau_{1}\right)}^{p1}}+\frac{R_{2}}{1+{\left(j\tau_{2}\right)}^{p2}}$$

where τ_1_ = (*R*1*T*1)^*p*1^ and τ_2_ = (*R*_2_ *T*_2_)^*p*2^

On fitting the proposed equivalent circuit, the exponents P₁ and P_2_ were found to be close to 1 when Rs was set to 0 Ω in SCAPS‐1D. However, impedance spectroscopy simulation revealed that the actual series resistance Rs is not zero, but rather ≈0.0006 Ω. In this scenario, the constant phase element (CPE) behaves as an ideal capacitor (T = C), causing the Cole–Cole relaxation^[^ ^84^ ^]^ to reduce to the Debye‐type relaxation (see Figure 18a). It's important to highlight that Debye relaxation is characterized by a single relaxation time (τ). However, according to experimental observations, the parameters p₁ and p_2_ in the exponent are typically less than 1, reflecting a distribution of relaxation times (τ). As a result, the total complex impedance of the circuit simplifies to Equation 21:

(21) $$Z^{*}\left(\omega \right)=Rs+\frac{R_{1}}{1+\left(j\omega \tau_{1}\right)}+\frac{R_{2}}{1+\left(j\omega \tau_{2}\right)}$$

Here,τ_1_ = R_1_C_1_ and τ_2_ = R_2_C_2_

According to ELMELOUKY et al (2024),^[^ ^117^ ^]^ the expressions for the angular frequencies (ω_1max_) and (ω_2max_) can also be derived from the derivative of the imaginary part (M″) with respect to angular frequency (ω). This results in obtaining the same specific value (ω_max_) corresponding to each maximum observed in the evolution of the imaginary part (M“”). With, $\;\omega 1max=\frac{1}{\tau_{1}}=\frac{1}{R_{1}C_{1}},\;\frac{1}{\tau_{2}}=\omega 2max=\frac{1}{R_{2}C_{2}}\;$. Therefore, the expressions help clarify why each peak observed in the evolution of the imaginary components of (Z″) and (M″) at both low and high frequencies appears at the same specific frequency value (F_max_).

### 4.5 Examination of the Parameters Derived from the Equivalent Circuit

**Figure 22:**
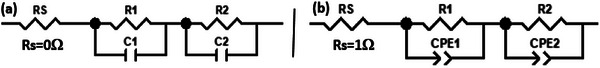
a) The electrical circuit modelling the solar cell Rs = 0 Ω and b) the circuit modelling the solar cell at Rs = 1Ω.

The equivalent model electrical circuit that represents the various spectra we previously plotted is as follows. **Figure** **22**, an ideal cell (Rs = 0) is represented by equivalent circuit (a). For a cell with electrode contacts that ensure charge collection (Rs = 1Ω), it is modelled by equivalent circuit presented in Figure 22b.

**Figure 23:**
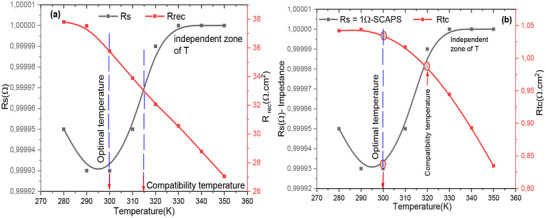
Variation of the a) series resistance Rs and R_rec_, of the b) series resistance Rs and R_tc_.

**Figure** **23**a,b shows the simultaneous evolution on a dual‐axis to facilitate a comparative study between the series resistance and the recombination resistance on one hand, and the series resistance and the charge transport resistance of the cell as a function of temperature on the other hand. It can be observed in both figures that the series resistance exhibits three distinct behaviors depending on the frequency: in the first zone, at low temperatures, it decreases until it approaches ambient temperature. The second zone shows an increase until it becomes independent of temperature, indicating that the series resistance is highly sensitive to the operating temperature of the cell. The recombination resistance and charge transport resistance both decreases. It is observed that the recombination resistance decreases in a linear fashion with temperature. However, for the charge transport resistance, it decreases almost linearly above a temperature of 290K.

**Figure 24:**
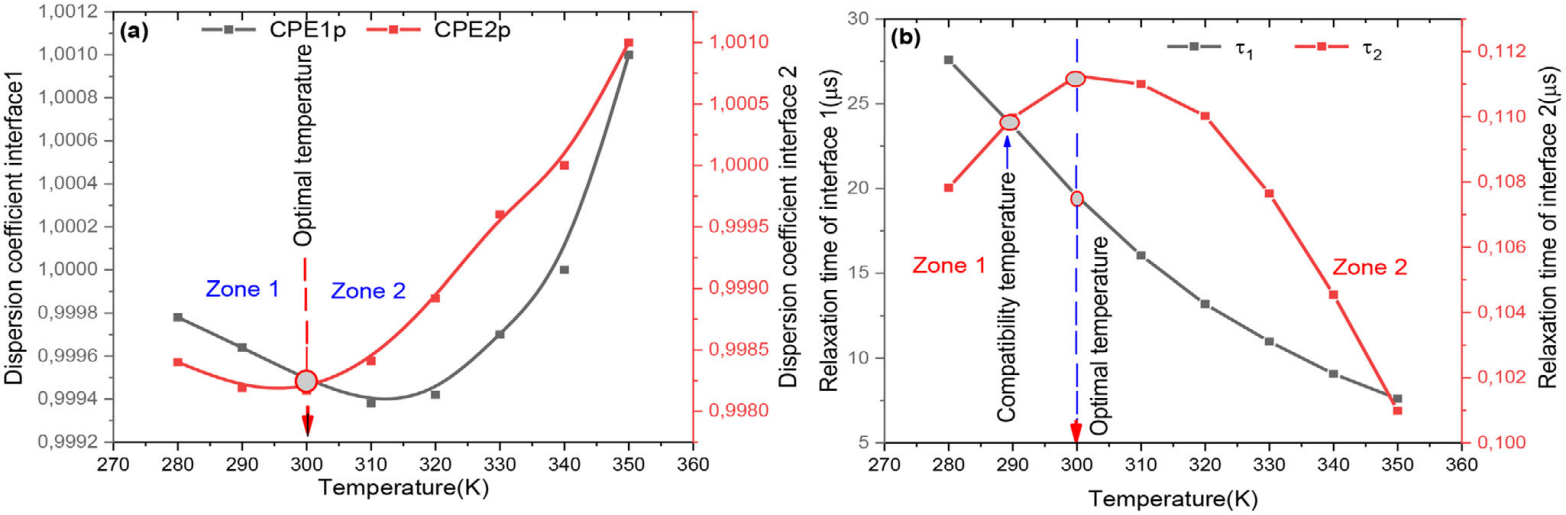
Variation of a) pseudo‐capacitance and b) relaxation time for interfaces 1 and 2.

**Figure** **24**a,b shows the variation of the dispersion coefficients of the interfaces and the relaxation times for interface 1 (WO_3_/N719 Dye) and interface 2 (N719 Dye/GO), respectively. We observe that, according to the analysis of the equivalent model circuit presented in Figure 23b, the operation of the cell as a function of temperature presents two distinct zones. One at low frequency, where the coefficients decrease as the temperature increases until the optimal temperature is reached. However, beyond the optimal temperature, the values of the dispersion coefficients increase linearly. This observation allowed us to measure the degree of homogeneity of the cell and the compatibility between the different layers constituting the cell. Figure 23b also shows that the operation of the cell exhibits two clearly identified zones: one at low temperature and the other at high temperature. Furthermore, both figures show a sharp change, indicating the optimal operating temperature of the cell.

## 5 Study of Activation Energy

**Activation energy** is a key parameter for understanding charge transport mechanisms in semiconductor materials, particularly in thin films. It represents the energy barrier that charge carriers (electrons or holes) must overcome to move through the material. In thin films, this energy is strongly influenced by the crystalline structure, defects, interfaces, and the nature of the deposition process. Studying the activation energy therefore provides valuable insights into thermal or electrical conduction processes and helps assess the material quality, especially in optoelectronic devices such as solar cells. After using the equivalent electrical circuit shown in Figure 21b, we obtained the relaxation frequencies summarized in **Table** **4**.

**Table 4:** Summary of the relaxation frequencies for each temperature.

Temperature [K]	Relaxation Frequency [Hz]	1000 [TfK‐1]	Ln(Fr)
280	6509,6	3,571	8,781
290	7940,3	3,448	8,979
300	8000	3,330	89,871
310	10 000	3,225	9,210
320	12 589	3,125	9,440
330	15 849	3.030	9,670
340	18 000	2,941	9,798
350	19 550	2,857	9,880

The relaxation frequency Fr follows a thermally activated behavior described by the Arrhenius law, expressed by the following equation^[^ ^113^ ^]^

(22) $$f_{r}=f_{0}\cdot \exp\left(-\frac{E_{a}}{k_{B}T}\right)$$

**Figure 25:**
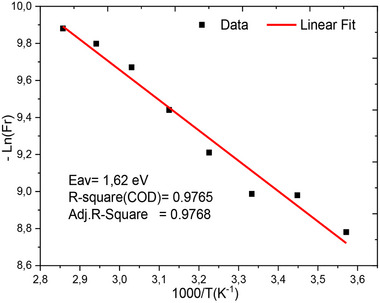
Arrhenius plot of the relaxation frequency.

Using Equation 22, we plotted the relaxation frequency as a function of 100/T. **Figure** **25** shows the Arrhenius plot of the relaxation frequency, extracted through modelling using the equivalent electrical circuit, in order to determine the activation energy of the studied cell.

Our analysis yielded an activation energy of 1.60 eV, which is comparable to the band gap of hybrid perovskites such as MAPbI_3_ (≈1.6 eV). In contrast, low‐bandgap perovskites used in tandem configurations typically exhibit band gaps in the range of 1.3 to 1.4 eV.

## 6 Comparative Analysis

**Table** **5** evaluates multiple solar cell designs by analysing key performance metrics: V_oc_, J_sc_, FF, and PCE. Each configuration reflects unique layer compositions and material selections. The proposed model cell achieves the highest simulated PCE of 20.80%, surpassing other designs in theoretical performance. While these results are computational projections, they highlight promising pathways for improving efficiency through optimized material pairings and structural engineering.

**Table 5:** Comparative performance of other ssDSSC device structures from literature.

Configuration	Method	V_oc_ [V]	J_sc_ [mA cm^−2^]	FF [%]	PCE [%]	Ref.
FTO/PC61BM/N719/CuSCN/Au	Simul.	≈1.0	0.885	70.94	5.38	[119]
FTO/ZnOS/N719 dye/CTZSe/Au	Simul	0.8751	20.83	70.86	12.91	[120]
FTO/TiO_2_/N719 dye/CuI/Pt‐FTO	Expt.	0.512	4.88	0.610	1.52	[121]
FTO/WO_3_/N79 Dye/GO/C	Simul.	1.1055	22.23	84.65	20.80	This work

Given these findings, the current work presents a promising approach for high‐efficiency dye‐sensitized solar cells. Since the results are based on simulations, experimental validation is necessary. Further, optimizing the thickness of WO_3_ and the properties of GO at the interface could enhance charge transport and stability.

## 7 Conclusions

This study presents a ground‐breaking dye‐sensitized model solar cell, achieving an impressive power conversion efficiency (PCE) of 20.8% with an innovative FTO/WO_3_/N79 Dye/GO/C cell configuration, designed with affordability and sustainability as the main focus. The impact of temperature variation in PCE is significant with 21.67% at approximately 285 K and steadily declines to about 12.5% at ≈475 K, representing a decrease of ≈42%. This decrease may be ascribed to dye degradation and increased resistance of the cell due to thermal agitation of excitons. Further, this work has found that at high shunt resistance effectively suppresses leakage currents and supports more efficient charge separation and collection, resulting in better photovoltaic outcomes – PCE, V_oc_, J_sc_ and FF. The N719 Dye active layer has a significant influence on the overall resistance of the cell because, as the cell's resistance increases, the power lost due to Joule heating also increases, leading to a reduction in the cell's power output. It is also noticeable that the presence of the N719 Dye absorber has a considerable effect when the Rs resistance increases. It is evident from this study that the frequency range (100 Hz–10 MHz) under study is highly efficient, and it can be deduced that the frequency range where the cell operates is the active zone. To understand the conduction mechanism in this cell structure during the intercalation of N719 dye, we observe very significant improvements at the cell interfaces. To analyze the most significant contribution, we used the Nyquist plot applied to the modulus function M∗. However, due to the roughness and heterogeneity of the interfaces formed between the different layers, the capacitor is generally replaced by a constant phase element (CPE).

## Conflict of Interest

The authors declare no conflict of interest.

## Author Contributions

G.G.N.: Conceptualization, writing original draft, Method development, Data analysis & Editing, A.E.: Method development, Supervision, Numerical analysis, writing & editing, E.L.M.: Method development, Supervision & Editing, N.R.: Simulation, Writing original draft, Analysis & Editing, J.K.K.: Conceptualization, Method development, Supervision, Simulation & Editing. All authors have read and approved the manuscript.

## Data Availability

The data that support the findings of this study are available from the corresponding author upon reasonable request.
